# Medication adherence with fixed-dose *versus* free-equivalent combination therapies: Systematic review and meta-analysis

**DOI:** 10.3389/fphar.2023.1156081

**Published:** 2023-03-22

**Authors:** Qiran Wei, Jiting Zhou, Hongchao Li, Luying Wang, Yao Wu, Aixia Ma, Xin Guan

**Affiliations:** ^1^ School of International Pharmaceutical Business, China Pharmaceutical University, Nanjing, Jiangsu, China; ^2^ Center for Pharmacoeconomics and Outcomes Research of China Pharmaceutical University, Nanjing, Jiangsu, China

**Keywords:** adherence, compliance, fixed-dose combination, free-equivalent combination, systematic review, meta-analysis

## Abstract

**Objective:** We conducted a large-scale meta-analysis and subgroup analysis to compare the effect of fixed-dose combination (FDC) therapy with that of free-equivalent combination (FEC) therapy on medication adherence.

**Methods:** Studies published in Web of Science, PubMed, Cochrane Library, ScienceDirect, and Embase up to May 2022 were identified according to Preferred Reporting Items for Systematic Reviews and Meta-Analyses (PRISMA) guidelines. The primary assessed outcomes were the medication possession ratio (MPR) and proportion of days covered (PDC). We investigated the probability of being adherent to the prescribed treatment (MPR or PDC ≥80%) or the average estimate of these two parameters. Studies reporting such results were included in this meta-analysis. The summary measures were reported as the risk ratio (RR) and the weighted mean difference (MD) with 95% of confidence interval (CI) using the random-effects model of DerSimonian and Laird. The quality of the cohort studies was assessed using the Newcastle-Ottawa scale.

**Results:** Of the 1,814 screened studies, 61 met the predefined inclusion criteria. The meta-analysis of the results showed that compared to FEC, FDC significantly improved the medication compliance of patients by 1.29 times (95% CI:1.23–1.35, *p* < 0.00001). I^2^ of 99% represent high heterogeneity across studies. The mean difference in medication adherence between FDC and FEC was 0.10 (95% CI: 0.06–0.14, *p* < 0.00001) with an I^2^ estimate of 100%. Subgroup analyses were performed for studies that reported adherence outcomes according to disease type, period of evaluation and compliance indicators. A sensitivity analysis was conducted to exclude the results of low-quality studies, as well as studies in which there was ambiguity in the method of calculating the estimator.

**Conclusion:** Analysis of the assessed parameters for the intention-to-treat and subgroup populations suggests that FDC can improve adherence to treatment and its advantages over FEC may increase over time. Further research is needed to better understand how medical conditions affect the impact of reduced pill burden on adherence, particularly in diseases other than cardiovascular disease and type 2 diabetes mellitus.

## 1 Introduction

Medication adherence, also known as compliance, is defined as “the extent to which a patient takes medication at prescribed intervals and dosages” (Cramer et al., 2008), and is usually measured over a given time period and reported as a percentage. Prospective studies can use electronic monitoring devices to obtain adherence data, while retrospective studies most widely use the medication possession ratio (MPR) and proportion of days covered (PDC) to calculate adherence to medication (Cramer et al., 2008; Raebel et al., 2013; Pednekar et al., 2019). In general, MPR or PDC is calculated as an average value; participants with MPR or PDC ≥80% are defined as “highly adherent” (Gellad et al., 2017). Contrary to high adherence, poor medication adherence is a pervasive medical challenge, with approximately half of chronically ill people not taking their medications as prescribed (Brown and Bussell, 2011). Not only does poor adherence results in limited effect of disease control, but it also imposes a huge disease burden on the healthcare system due to possible associated complications and adverse events (Cutler et al., 2018). The multifactorial causes of reduced compliance are classified into three categories: patient-related factors, physician-related factors, and healthcare team and system-related factors (Brown and Bussell, 2011). Evidence suggests that patient adherence is positively associated with expected or actual efficacy (e.g., in human immunodeficiency virus disease, cancer, and gastrointestinal disease), and is negatively associated with protocol complexity (e.g., in lung disease, diabetes, and sleep disturbances) (DiMatteo, 2004).

Polypharmacy (the use of multiple drugs simultaneously) is a significant issue in clinical practice, and it is becoming increasingly prevalent worldwide. For example, the number of U.S. adults aged 20 or older who were prescribed five or more drugs increased from 8.2% in 1999%–2000% to 15% in 2011–2012 (Masnoon et al., 2017; Guillot et al., 2020). Considering the need of combination therapy for certain diseases and patients with comorbidities, most patients need to take two or more drugs for the best treatment outcomes. Thus, many people may simultaneously have chronic cardiovascular diseases such as hypertension, hyperlipidaemia or/and diabetes, which means that besides the antihypertensive treatment (Kalra et al., 2010), they also need to take drugs for these other comorbidities. People with chronic diseases do not experience symptoms of illness on the short term, which makes them more likely to forget taking their medicine. These complex and long-term medication patterns pose great challenges to medication adherence. The use of fixed-dose combination (FDC) drugs, which combine two or more existing active substances into a single tablet, will simplify treatment regimens, reduce the pill burden, and potentially improve medication adherence (Cazzola and Matera, 2017; DiPette et al., 2019; Sarzani et al., 2022), contributing to substantial clinical and economic benefits. However, it is important to acknowledge that FDC may present some limitations, including the difficulty in adjusting the dose of individual components (LaFleur et al., 2006) and attributing adverse events to specific drugs (Schaffer et al., 2017). These limitations should be carefully considered in clinical practice to ensure the safe and effective use of FDC.

Recent studies have investigated the relationship between FDC and adherence improvement, showing that a reduction in the number of pills leads to better adherence (Kawalec et al., 2018; Baumgartner et al., 2020; Weisser et al., 2020). Kawalec et al. (2018) assessed the adherence to FDC and free-equivalent combination (FEC) therapies demonstrating the clinical advantages of FDC during the treatment of arterial hypertension. However, this meta-analysis analysed different measures of adherence (MPR vs PDC), ignoring the impact of different evaluation periods. A meta-analysis was performed by Weisser et al. (2020) compared the efficacy of FDC *versus* FEC therapy in patients with hypertension, dyslipidaemia, or a combination of the two diseases, focusing on studies with a PDC ≥80%. The results confirmed better medication adherence with FDC therapy, but different periods of evaluation were not considered. Baumgartner et al. (2020) summarised the evidence on the effect of FDC on medication compliance in original peer-reviewed systematic reviews, but they did not conduct a meta-analysis because the included articles used 11 different compliance indicators, which made it difficult to compare the study results.

Therefore, we conducted a comprehensive analysis of the impact of FDC therapy on medication adherence across a wide range of diseases, focusing specifically on studies that used two compliance indicators and accounting for different evaluation periods. The results of the comparison will provide theoretical support for policymakers, clinicians and patients in choosing treatment methods.

## 2 Materials and methods

### 2.1 Search strategy

This systematic review was conducted in accordance with the Preferred Reporting Items for Systematic Reviews and Meta-Analyses (PRISMA) guidelines (Page et al., 2021a; Page et al., 2021b). We conducted a systematic literature search for original articles published up until May 2022 using five electronic databases: Web of Science, PubMed, Cochrane Library, ScienceDirect, and Embase. We used a search strategy that included combinations of MeSH and free-text terms: “adherence”, “compliance”, “fixed dose”, “single-pill combination”, “polypill”, “free dose”, “free combination”, “medication possession ratio”, “proportion days covered”, *etc.* A parallel search strategy was used for all databases. All literature was imported into NoteExpress software for data management. The PubMed database search strategy adapted to other databases is shown in Supplementary Table S1.

### 2.2 Eligibility criteria

Based on the PICOS (Population, Interventions, Comparison, Outcome and Study Design) format, the eligibility criteria for studies included in this systematic review were set as follows.

(1) Population: studies including patients diagnosed with any disease;
(2) Interventions and comparison: studies including FDC or FEC therapy as intervention and comparator;
(3) Outcome: studies in which compliance was reported as the primary outcome, using at least one index such as the PDC or MPR.
(4) Study design: all types of research studies, including both clinical trials and observational studies.

We excluded articles if they were 1) economic evaluations, 2) conference abstracts, editorials, study protocols, letters, or reviews, and 3) not in English language.

### 2.3 Study selection and data extraction

Two reviewers (Wei and Zhou) independently screened the literature, extracted and cross-checked the data. The reviewers performed a first screening by titles and abstracts and a second screening of the included articles by reading the full text for the final evaluation. In addition, we checked the references of the reviewed literature and traced them back to the original studies to acquire other potentially relevant studies. Articles that met the above mentioned criteria were eventually included in this study and information was extracted accordingly. In cases where disagreement appeared and could not be resolved, the viewpoint of the third researcher was considered. Information was extracted from the included studies using standardised data extraction tables as follows: 1) general information: first author, year of publication, country or region, demographic information (gender, age, and sample size); 2) study design: period of evaluation, diagnosis (“type of disease”), and dosage form; and 3) focused intervention, comparator, and the main results regarding adherence.

### 2.4 Quality appraisal

The two reviewers (Wei and Zhou) independently evaluated the quality of the identified studies. Disagreements were resolved by consensus or by a third researcher. The quality of the included studies was assessed using the Newcastle-Ottawa scale (for cohort studies) assigning a note from one to nine (Wells et al., 2014). The tool has eight items within three dimensions (patient selection, comparability of the study groups, and outcome assessment). A study with a score of six or higher was considered high quality.

Follow-up completeness is a pre-requisite for reliable outcome assessment. A sufficiently long follow-up period was defined to allow a reasonable percentage of follow-up loss to follow up the cohort appropriately (i.e., 1 year and 20%).

### 2.5 Statistical methods

The primary outcome of interest was medication adherence assessed by the MPR or PDC. The MPR is calculated as the ratio of the number of days’ supply obtained during a specified time period to the number of days within the prescription refill interval (Sperber et al., 2017). The PDC is calculated as the ratio of the number of days in which all medications were available to the patient to the total number of days in a given period (e.g., study period) (Prieto-Merino et al., 2021). We investigated the probability of being adherent to the prescribed treatment (MPR or PDC ≥80%) or the average estimate of these two parameters. Studies reporting such results were included in this meta-analysis.

Results were analyzed following Intention-to-Treat (ITT). We used as effect measures the risk ratio (RR) for dichotomous data and the weighted mean difference (MD) for continuous data. We investigated the probability of being adherent to the prescribed treatment and studies not using 80% as a cut-off value were not included in the meta-analysis. The number of patients who were adherent to the treatment was either already given in the original publication or calculated using the total number of patients in each treatment group and the corresponding percentage who were adherent. All mean and standard deviation values were obtained from the original article. The 95% confidence interval (CI) was calculated for both the FDC and FEC groups. Heterogeneity was assessed using the I^2^ statistic. If the results showed significant heterogeneity (I^2^ ≥ 40%), a random-effects model of DerSimonian and Laird was used; otherwise, a fixed-effects model was used. We also examined differences in the period of evaluation, disease type, compliance indicators, and pharmaceutical form of drugs between studies to investigate sources of heterogeneity and reveal possible subgroups. If there were less than three studies in a subgroup, no meta-analysis was done. Sensitivity analysis was further performed to exclude low quality studies and studies which did not clearly define the method of calculating the estimator. At the same time, for results that could not be quantitatively analysed, we present the findings in narrative form and appropriate tables, for a comprehensive presentation of the data.

All data analyses were performed using the RevMan 5.4.1 software (Copenhagen: The Nordic Cochrane Centre, the Cochrane Collaboration, 2020) and double data entry to avoid input errors.

## 3 Results

### 3.1 Study selection

**FIGURE 1:**
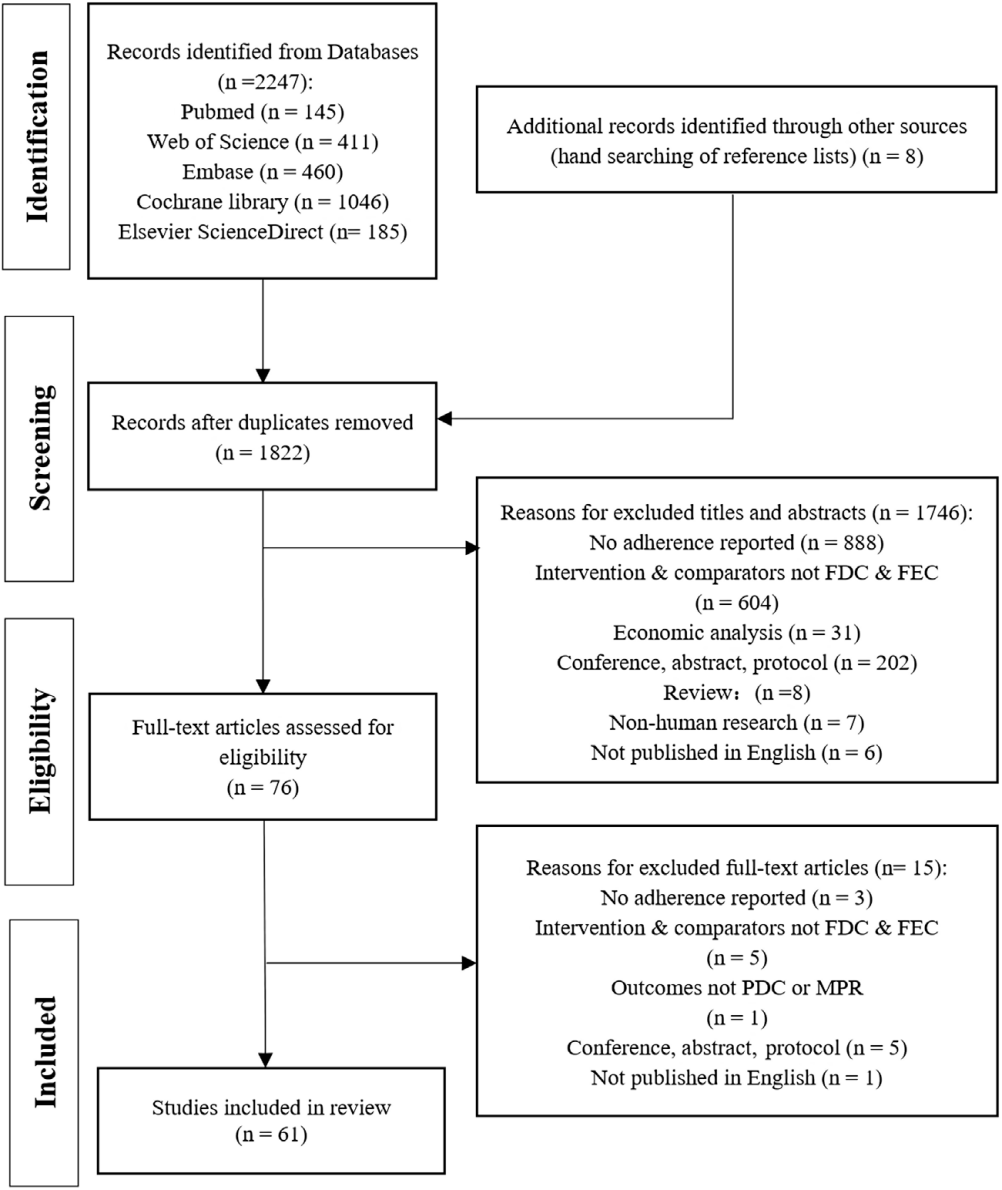
PRISMA flow diagram for study selection process. FDC: fixed-dose combination; FEC: free-equivalent components.

The database search yielded 2,247 records of which 1,814 remained after removing duplicates. After the screening of the titles and abstracts, 76 potentially relevant studies were selected, and after full-text screening, 61 articles were included. Figure 1 shows the flow of article selection and the reasons of study exclusion.

### 3.2 Characteristics of the included studies

A total of 61 studies were finally included for further consideration; their characteristics are shown in Supplementary Table S2. Studies were conducted in different countries, and more than half of them were conducted in the United States (n = 37). The proportion of men in these studies and their mean age ranged from 17.3% to 97.4% and from 42.0 to 76.6 years, respectively. All of studies were retrospective. Hypertension (n = 34) was the most frequent disease type in the 61 included studies (Taylor and Shoheiber, 2003; Gerbino and Shoheiber, 2007; Dickson and Plauschinat, 2008a; Dickson and Plauschinat, 2008b; Brixner et al., 2008; Hess et al., 2008; Jackson et al., 2008; Patel et al., 2008; Chapman et al., 2009; Shaya et al., 2009; Hussein et al., 2010; Yang et al., 2010; Zeng et al., 2010; Baser et al., 2011; Breitscheidel et al., 2012; Ferrario et al., 2013; Panjabi et al., 2013; Baggarly et al., 2014; Degli et al., 2014; Wang et al., 2014; Xie et al., 2014; Hsu et al., 2015; Machnicki et al., 2015; Tung et al., 2015; Levi et al., 2016; Saito et al., 2016; Lauffenburger et al., 2017; Bramlage et al., 2018; Ho et al., 2018; Verma et al., 2018; Ah et al., 2019; Kim et al., 2019; Wang et al., 2019; Choi et al., 2021), followed by diabetes (n = 9) (Melikian et al., 2002; Vanderpoel et al., 2004; Cheong et al., 2008; Pan et al., 2008; Thayer et al., 2010; Barner, 2011; Vittorino et al., 2014; Lokhandwala et al., 2016; Bohm et al., 2021), acquired immune deficiency syndrome (AIDS) (n = 5) (Kauf et al., 2012; Tennant et al., 2015; Scott et al., 2016; Sutton et al., 2016; Yager et al., 2017), hyperlipidaemia (n = 2) (LaFleur et al., 2006; Bartlett et al., 2017), and dyslipidaemia (n = 2) (Kamat et al., 2011; Rea et al., 2021). Other disease types included each in one article were lower urinary tract symptoms (AH et al., 2020), benign prostatic hyperplasia (Eisen et al., 2020), lower urinary tract symptoms with benign prostatic hyperplasia (Drake et al., 2017), chronic obstructive pulmonary disease (COPD) (Mannino et al., 2022), Parkinson’s disease (Delea et al., 2010), glaucoma (Shirai et al., 2021), asthma (Haupt and Nilsson, 2010), mixed hyperlipidaemia/hypertension (Schaffer et al., 2017), and cardiovascular disease (Balu et al., 2009). The number of patients included was reported in all studies and ranged from 48 to 579,851. Based on the Newcastle–Ottawa Scale Quality assessment scale, 48 included studies were of high quality, while 13 studies were classified as low quality. The average quality score of the 61 studies was 6.70. The specific rating scores are listed in Supplementary Table S3, and the details are shown in Supplementary Table S4.

### 3.3 Adherence outcome

In our analysis, 61 studies provided information on MPR or PDC, with periods of evaluation ranging from 3 months to 5 years. The common cut-off value for MPR or PDC was 80%. Additionally, most studies obtained adherence data through assessment of electronic databases, except for the study on AIDS by Tennant et al. (2015), which included both pharmacy-filling records and self-reported outcomes. In three articles, the drugs studied (Haupt and Nilsson, 2010; Shirai et al., 2021; Mannino et al., 2022) were not taken orally; therefore we did not include them in the meta-analysis because of the possible bias in adherence owing to different dosage forms. The measurement methods and more outcomes’ details are presented in Supplementary Table S3.

#### 3.3.1 Adherence in the ITT population

**FIGURE 2:**
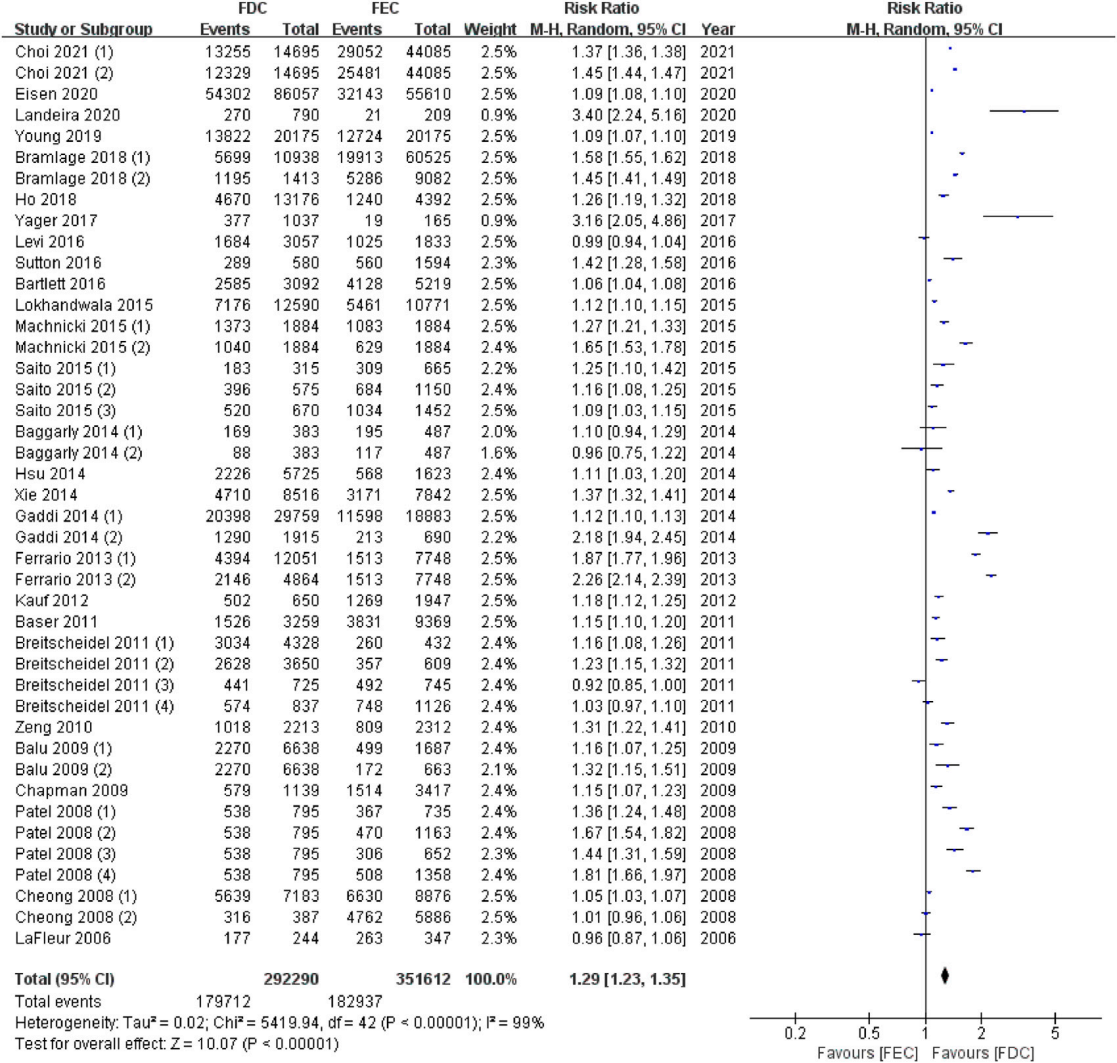
Meta-analysis of risk ratio for medicine high adherents (MPR or PDC ≥ 0.8) in ITT population. MPR: medication possession ratio; PDC: proportion of days covered; FDC: fixed-dose combination; FEC: free-equivalent components; ITT: Intention-to-treat analysis.

**FIGURE 3:**
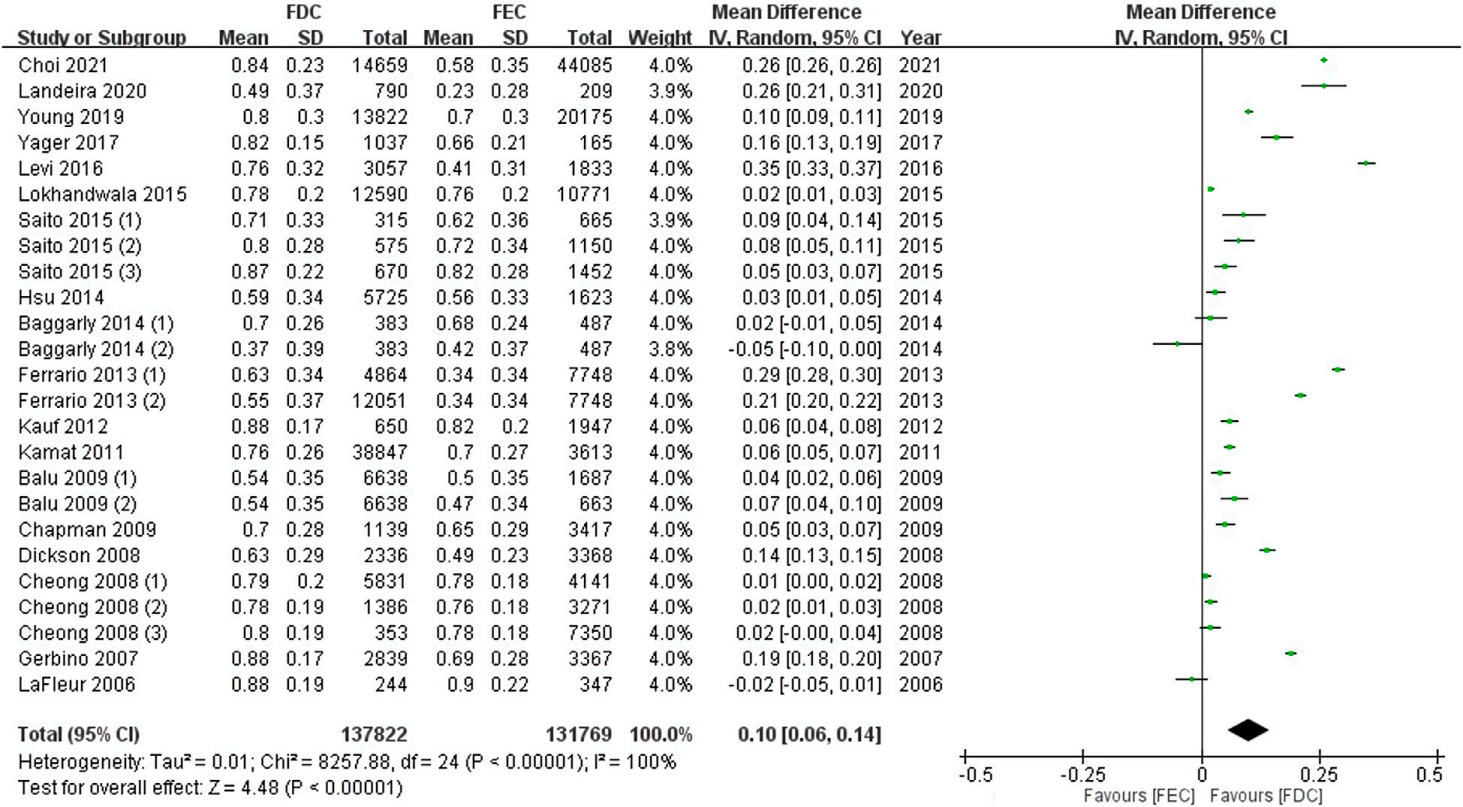
Meta-analysis of weighted mean difference in MPR or PDC in ITT population. MPR: medication possession ratio; PDC: proportion of days covered; FDC: fixed-dose combination; FEC: free-equivalent components; ITT: Intention-to-treat analysis.

In total, 27 articles reported data on adherent patients (MPR or PDC ≥0.8), and 18 studies provided information on the average MPR or PDC in each group. For ITT population, the meta-analysis using a random model showed that, compared with FEC, FDC significantly improved the medication compliance of patients by 1.29 times (95% CI: 1.23–1.35, *p* < 0.00001, Figure 2). I^2^ of 99% represent high heterogeneity across studies. Likewise, the mean difference in medication adherence between FDC and FEC was 0.10 (95% CI: 0.06–0.14, *p* < 0.00001, Figure 3) with an I^2^ estimate of 100%.

#### 3.3.2 Adherence in subgroup population

Subgroup analyses were performed for studies that reported adherence outcomes by disease type (diabetes vs. cardiovascular disease), period of evaluation (3 months vs. 6 months vs. 12 months) and compliance indicator (MPR vs. PDC).

In total, 22 cohort studies showed the FDC therapy was associated with the high medicine adherence in type 2 diabetes mellitus (T2DM) and cardiovascular disease (including hypertension, hyperlipidaemia, dyslipidaemia, and mixed hyperlipidaemia/hypertension). The meta-analysis demonstrated that in patients with T2DM (3 studies) and cardiovascular disease (19 studies), the adherence to treatment was 19% and 28% higher, respectively, in the FDC group than in the FEC group (*p* < 0.00001, Supplementary Figure S1). I^2^ of 98% and 99% shows significant heterogeneity between studies. In addition, 16 cohort studies reported the mean MPR or PDC related to the therapy of these two diseases and the corresponding standard deviation. The mean difference of medication adherence for FDC vs. FEC therapy was 2% and 11% for patients with T2DM and cardiovascular disease (95% CI: 0.01–0.02, *p* < 0.00001 and 95% CI: 0.07–0.16, *p* < 0.00001, Supplementary Figure S2). I^2^ of 39% demonstrate low heterogeneity across T2DM studies and I^2^ of 100% demonstrate significant heterogeneity across studies of cardiovascular disease.

A meta-analysis of six cohort studies demonstrated that the percentage of adherent patients was significantly higher for patients on FDC treatment than for those on FEC treatment after 6 months of therapy (pooled RR = 1.32; 95% CI: 1.18–1.47; *p* < 0.00001); this percentage slightly increased after 12 months (pooled RR = 1.34; 95% CI: 1.23–1.47, Supplementary Figure S3). Likewise, in the meta-analysis based on the mean MPR or PDC, compared to FEC, significant advantages were seen for FDC at 12 months (MD = 0.10, 95% CI: 0.04–0.15, *p* < 0.00001), not at 3 and 6 months (Supplementary Figure S4). Meta-analyses by period of evaluation show a heterogeneity close to 100%.

16 studies have reported that FDC therapy was associated with high medication adherence as measured by MPR (MPR ≥80%), while 12 studies have reported high adherence as measured by PDC (PDC ≥80%). Among these, one study utilized both MPR and PDC to measure compliance (Machnicki et al., 2015). The meta-analysis demonstrated that the MPR in the FDC group was 20% higher, while the PDC was 39% higher, than in the FEC group (*p* < 0.00001, Supplementary Figure S5). The high I^2^ values of 99% and 98% indicate significant heterogeneity between the studies. Furthermore, 13 cohort studies reported the mean MPR, while five cohort studies reported the mean PDC. The mean difference in medication adherence for FDC *versus* FEC therapy was 9% and 13% for the two compliance indicators, respectively (95% CI: 0.05–0.13, *p* < 0.00001 and 95% CI: 0.04–0.23, *p* < 0.00001, Supplementary Figure S6). Meta-analyses by adherence indicators also show a heterogeneity close to 100%.

#### 3.3.3 Sensitivity analysis

A sensitivity analysis was conducted to exclude the results of low-quality studies, as well as studies in which there was ambiguity in the method of calculating the estimator. 23 high-quality articles reported data on adherent patients (MPR or PDC ≥0.8), while 14 high-quality studies provided information on the average MPR or PDC. Eleven articles in total had unclear or confused definitions of MPR or PDC (Dickson and Plauschinat, 2008a; Hess et al., 2008; Pan et al., 2008; Shaya et al., 2009; Thayer et al., 2010; Baggarly et al., 2014; Saito et al., 2016; Verma et al., 2018; Ah et al., 2019; Wang et al., 2019; AH et al., 2020).

The meta-analysis using a random model showed that, compared with FEC, FDC significantly improved the medication compliance of patients by 1.29 times (95% CI: 1.22–1.36, *p* < 0.00001, Supplementary Figure S7). High heterogeneity across studies was observed with an I^2^ of 99%. Additionally, the mean difference in medication adherence between FDC and FEC was 0.08 (95% CI: 0.02–0.15, *p* < 0.00001, Supplementary Figure S7) with an I^2^ estimate of 100%.

## 4 Discussion

This study aimed to compare the impact of FDC and FEC on medication adherence by reviewing the current literature. Using the Newcastle-Ottawa scale, we evaluated that the 61 studies included herein were of high quality, with an average score of 6.7. Our analyses on adherence in ITT population and subgroup population suggested that the use of a single tablet combining multiple active substances could improve adherence to treatment regimens. While acknowledging the limited evidence currently available on pathologies beyond hypertension and T2DM, our analysis highlights the potential benefits of the FDC strategy in improving medication adherence across a broad range of chronic diseases. We believe that further research in this area is warranted to fully explore the potential of FDC as a therapeutic approach for these conditions.

In recent years, studies focusing on the advantages of FDC, mainly in the field of chronic diseases, demonstrate its importance. FDC treatment is associated with a significant improvement in adherence to the treatment of single diseases, such as hypertension (Sherrill et al., 2011; Kawalec et al., 2018; Weisser et al., 2020; Parati et al., 2021) and T2DM (Han et al., 2012). To the best of our knowledge, our study represents the first comprehensive systematic review and meta-analysis of the benefits of FDCs in improving medication adherence. Our analysis includes a wide range of original peer-reviewed studies spanning multiple disease types, making it the most extensive examination of this topic to date. Furthermore, our study is based on a rigorous selection of available studies which was performed to minimise bias. Only studies with outcomes clearly assessed using MPR or PDC were considered eligible.

We conducted subgroup analyses for studies that reported adherence outcomes considering the disease type, duration of the study and compliance indicator. For hypertension and T2DM, the compliance was significantly higher in the FDC group than in the FEC group; these results were in concordance with those of earlier systematic reviews (Sherrill et al., 2011; Du et al., 2018; Kawalec et al., 2018). In addition, our study found that, the percentage of adherent patients and mean MPR/PDC was significantly higher for those on FDC treatment than for those on FEC treatment after 6 months of therapy. This advantage of FDCs over FECs in terms of medication adherence may become more apparent over time. However, limited number of studies reporting mean MPR or PDC at 3 or 6 months could increase the instability of the results and prevent us from reaching definitive conclusions. Further studies are needed to confirm this hypothesis. Besides, the findings of this study indicated that PDC might be higher than MPR in the evaluated patient population. Therefore, a comprehensive assessment of medication adherence should include both MPR and PDC or other measures to obtain a more accurate picture of medication adherence.

As patients tend to be highly compliant in clinical trials, data from real-world settings are more suitable for adherence studies. Thus, all the studies included in our systematic review were real-world studies. For most of them, data were obtained from databases. Only one article provided adherence data from dual sources: pharmacy-filling records and patient self-reports. This study showed that the average patient-reported adherence rates were higher than those recorded in outpatient clinics (Tennant et al., 2015), likely owing to recall and interview biases. In contrast, indirect assessments through claims databases and other means may underestimate or overestimate the level of compliance of patients. This is clear because databases comprising the medication status of patients cannot fully and effectively reflect reality: for example, patients may have obtained medication from other sources or been prescribed a medication, but they are not taking it.

Most of the included articles indicated that single-pill therapy had a positive effect on treatment compliance, but some articles suggested that FDC was not significantly better than FEC. Two studies on AIDs (Tennant et al., 2015; Yager et al., 2017) concluded that there was no significant correlation between a better adherence and the use of FDC therapy. This might be due to the selection of patients and a limited sample size. The study by Lafleur et al. (2006) further indicated that this might be due to possible drug switching in the FEC group, which is common in clinical practice; however, in the FDC group the switching behaviour was not an option. Thus, we must acknowledge a potential drawback of combination therapy: therapy with FDC may be less flexible than that with FEC. When adverse events occur, it is difficult for doctors to determine which active substance is responsible, resulting in unnecessary treatment and extra medical costs for patients and the medical insurance system. Furthermore, an insufficient number of participants or short follow-up periods may have also prevented the detection of statistically significant differences between groups (Buscher et al., 2012; Matsumura et al., 2012). Some polypharmacy therapies do not have high drug burden indexes; thus, their medication strategy is not associated with adherence (Buscher et al., 2012).

Although the PDC and MPR have been widely recognised in the literature, they have inherent limitations. A cut-off value of 80% may not adequately discern clinically adherent behaviour. Specifically, adherence defined as a MPR of either 79% or 81% was associated with similar treatment costs and outcomes, but only the patient group with an MPR of 81% was considered adherent. To minimise this bias, the mean value was used as an effect measure in our review.

Additionally, medication adherence is a dynamic process, and PDC and MPR may not be able to sensitively capture changes in patient behaviour over time. This dynamic process is influenced by a variety of factors related to patients, prescriptions, doctor-patient communication, socioeconomics, and the healthcare system (Brown and Bussell, 2011). The general use of combination therapy and quantified adherence with established metrics, ignoring the variability of medicine-taking behaviours and potential differences behind different behaviours, can lead to missed opportunities to address barriers which impair appropriate medication use. Compared to the above-mentioned metrics, group-based trajectory modelling can describe behaviour over time. For instance, in a study by Nicholas et al. (2022), 39.5% of patients with multiple sclerosis were non-adherent to oral disease-modifying treatment. While conducting this analysis, they found three patterns of adherence: the “immediate non-adherence”, the “gradual non-adherence”, and the “adherence” group. These analyses suggest that different patients have different adherence patterns, and they might require different types of clinical interventions. Exploring different adherence patterns will facilitate the better utilisation of medical resources and improve patient outcomes.

During systematic screening and article extraction, we found that some articles used a combination of “compliance”, “adherence”, and “persistence”. In some studies, the duration of treatment up to discontinuation was defined as an incorrect measure of adherence. Medication persistence is defined as “the act of continuing treatment from initiation to cessation of the prescribed duration”. No one uniform term that can combine these three distinct concepts (Cramer et al., 2008). In addition, although MPR and PDC are commonly used measures of medication adherence, they have distinct definitions and calculation methods. We found some ambiguity regarding the calculation of these indicators in our included studies, which may have contributed to the observed heterogeneity in our meta-analysis. Accordingly, we analyzed the indicators separately in our subgroup analysis, and then excluded in a sensitivity analysis the results of the studies for which there is ambiguity in the method of calculating the estimator. To improve the comparability of future studies and avoid any misunderstanding of the study by the reader, future research should take care to clearly define and differentiate the corresponding terms and measuring methods.

Our study has several limitations that should be considered when interpreting the results. First, most meta-analyses show a heterogeneity close to 100%, and high heterogeneity reduces the certainty of the results. The selected observational studies are characterized by large differences in the design. Variations in health systems, diagnoses, cultural backgrounds, and population characteristics across countries also have an impact on research outcomes. In addition, our study did not incorporate all articles on adherence. The review is limited to English, which may be biased by the exclusion of some articles published in other language. Moreover, we did not include studies in which the definition of terms was not uniform and measured parameters different from PDC or MPR. Although some articles reported PDC or MPR, they were also not included in the meta-analysis because these parameters did not use 80% as a cut-off value and no standard deviation was reported. In the future, we will obtain more data by contacting relevant researchers and attempt to identify the potential confounding variables for a more in-depth and comprehensive analysis. Another limitation is that we only analyzes medication adherence without exploring its potential impact on clinical outcomes. Although understanding the relationship between adherence and clinical outcomes could provide further insights into the implications of adherence on treatment outcomes, we did not make this the focus of our study. The effect of adherence on clinical outcomes needs to be compared by the same metrics within the same disease area to draw definitive conclusions. It is worth noting, however, that previous studies have explored the relationship between adherence and clinical outcomes for diseases such as diabetes (Katsiki et al., 2020) and hypertension (Parati et al., 2021), and further research in this area may provide valuable insights.

## 5 Conclusion

Analysis of the ITT and subgroup populations suggests that FDC therapy is associated with an improvement in patients’ adherence to the treatment schedule, and its advantages over FEC may increase over time. Further research is needed to better understand how medical conditions affect the impact of reduced pill burden on adherence, particularly in diseases other than cardiovascular disease and T2DM.

## Data Availability

The original contributions presented in the study are included in the article/Supplementary Materials, further inquiries can be directed to the corresponding authors.
