# Supplementary material for: Medication adherence with fixed-dose versus free-equivalent combination therapies: Systematic review and meta-analysis
Source: Front Pharmacol. 2023 Mar 22;14:1156081. doi: 10.3389/fphar.2023.1156081 (PMC10074603; doi:10.3389/fphar.2023.1156081)
Supplement: Supplementary file 1 [file DataSheet1.pdf]

Supplementary Material

Medication adherence with fixed-dose versus free-equivalent combination therapies: Systematic review and meta-analysis

Qiran Wei[1,2], Jiting Zhou[1,2], Hongchao Li[1,2], Luying Wang[1,2], Yao Wu[1,2], Aixia Ma[1,2]* and Xin Guan[1,2]*

*** Correspondence:**

Aixia Ma: ma86128@sina.com

Xin Guan: gg_cpu@163.com

1 Supplementary Tables

Table S1 A PubMed database search strategy

Search	Query	
#1	((fixed dose) OR (fixed-dose) OR (multiple-pill combination) OR (free combination) OR (fixed combination) OR (free-equivalent combination) OR (free dose) OR (polypill) OR (multicap) OR (single-pill) OR (single pill) OR (double-pill) OR (double pill) OR (two-pill) OR (triple pill) OR (triple-pill) OR (triple combination)) OR (Drug Combination [MeSH Terms])	Fixed dose combination and free-equivalent combination therapy
#2	(adherence) OR (compliance) OR (persistence) OR (continuity)	Adherence/compliance
#3	(medication possession ratio) OR (proportion days covered) OR (MPR) OR (PDC)	Target terms
#4	#1 AND #2 AND #3	

Table S2 Characteristics of Included Studies

Study	Country/region	Male, %		Age, Mean (SD)		Disease	Dosage form	Sample number	Newcastle-Ottawa Scale rating
		FDC	FEC	FDC	FEC				
Mannino 2022	America	45.5	47.9	60.6 (7.8)	60.4 (7.8)	COPD	Inhalant	9,942	9
Bohm 2021	Germany	71.7	71.8	62.1	62.2	T2DM	Pill	990	7
Choi 2021	Korea	48.5	48.4	62.6 (11.7)	62.6 (11.8)	Hypertension	Pill	58,780	6
Rea 2021	Italy	62.7	61.6	Only numbers per age range are given		Dyslipidemia	Tablet	4,258	5
Shirai 2021	Japan	55.0	57.1	56.5 (10.2)	57.1 (10.2)	Glaucoma	Eye drops	638	9
Eisen 2020	Germany	/	/	76.4 (9.3)	76.6 (9.3)	BPH	Tablet	141,667	6
Landeira 2020	Spain	/	/	69.4 (9.8)	71.7 (9.5)	LUTS	Tablet	999	8
Kim 2019	Korea	56.3	52.6	56.9 (12.3)	61.1 (12.4)	Hypertension	Tablet	28,876	7
Wang 2019	America	44.4	40.7<br>54.8	70.7 (8.5)	72.5 (9.0)<br>72.3 (9.5)	Hypertension	Pill	10,366	4
Ah 2019	Korea	58.8	58.7	56.1 (13.8)	56.1 (13.9)	Hypertension	Pill	40,350	8
Bramlage 2018	Germany	56.2<br>49.5	49.9<br>43.8	63.4 (14.1)<br>64.5 (13.1)	68.9 (14.1)<br>70.0 (13.3)	Hypertension	Pill	81,958	6
Ho 2018	Taiwan	52.9	53.6	58.8 (13.5)	59.0 (13.8)	Hypertension	Pill	17,568	9
Verma 2018	Canada	46.2	44.9	/	/	Hypertension	/	13,350	9

Bartlett 2017	Australia	55.7	59.8	/	/	Hyperlipidemia	Tablet	9,391	7
Drake 2017	Netherlands	/	/	70.5 (9.2)	72.8 (9.7)	LUTS with BPH	Tablet	1,891	7
Lauffenburger 2017	America	48.0	63.6	49.3 (10.6)	52.5 (11.4)	Hypertension	Tablet or capsule	101,224	7
Schaffer 2017	Australia	47.6	47.0	Only numbers per age range are given		Hypertension and/or hyperlipidemia	Tablet	9,430	7
Yager 2017	America	95.8	97.4	54.0 (5.0)	50.0 (5.0)	AIDS	Tablet	1,202	5
Levi 2016	Italy	47.8	49.2	66.6 (12.6)	68.1 (11.6)	Hypertension	Pill	6,612	4
Saito 2016	Japan	48.6	49.3	68.8 (12.3)	67.7 (12.1)	Hypertension	Tablet	2,148	5
Sutton 2016	America	44.7	45.0	43.0 (10.0)	42.0 (10.0)	AIDS	Tablet	2,174	5
Sutton 2016	America	97.4		52.0		AIDS	Tablet	15,602	5
Lokhandwala 2015	America	61.9	63.0	54.5 (10.9)	55.3 (11.6)	T2DM	/	23,361	8
Machnicki 2015	America	51.6	52.0	66.4 (12.0)	66.8 (12.9)	Hypertension	Pill	3,768	7
Tennant 2015	America	61.8	75.9	/	/	AIDS	Tablet	389	6
Baggarly 2014	America	34.0		Only numbers per age range are given		Hypertension	/	870	8
Degli 2014	Italy	/	/	/	/	Hypertension	Tablet	104	6
Hsu 2014	Taiwan	55.9	54.4	55.0 (12.6)	55.9 (12.9)	Hypertension	/	7,350	7

Tung 2014	Taiwan	52.2	52.0	60.3 (12.5)	60.4 (13.1)	Hypertension	Pill	16,505	7
Vittorino Gaddi 2014	Italy	50.9		/		T2DM	Pill	51,247	6
Wang 2014	Taiwan	42.4		65.8 (12.7)		Hypertension	Tablet	896	7
Xie 2014	America	54.8	50.6<br>48.7	54.8 (11.0)	59.1 (11.9)<br>63.9 (12.1)	Hypertension	Pill	17,528	7
Ferrario 2013	America	58.1<br>56.2	50.8	53.8 (11.2)<br>56.0 (11.9)	60.7 (12.5)	Hypertension	Pill	24,663	8
		50.5	50.2	58.8 (11.2)	58.2 (12.0)			5,340	
Panjabi 2013	America	54.7	56.0	57.6 (11.5)	56.8 (11.9)	Hypertension	Pill	9,123	7
		41.2	41.8	60.3 (12.2)	59.8 (13.4)			1,827	
Kauf 2012	America	84.6	82.0	Only numbers per age range are given		AIDS	Tablet	2,597	7
Barner 2011	America	/	/	/	/	/	Tablet	60	7
Baser 2011	America	59.5	52.5	54.4 (10.7)	60.8 (11.9)	Hypertension	Pill	12,628	9
Breitscheidel 2011	Germany	/	/	/	/	Hypertension	Tablet	12,452	7
Kamat 2011	the United States	55.2	62.3	56.4 (12.3)	54.8 (11.6)	Mixed dyslipidemia	Tablet	42,460	7
Delea 2010	America	63.0	64.0	73.0 (9.0)	73.0 (9.0)	Parkinson’s disease	Tablet	1,211	9

Hussein 2010	America	/	/	/	/	Hypertension	Pill	35,430	8
Thayer 2010	America	61.7<br>63.7	64.1<br>64.3	54.7 (10.2)<br>55.3 (9.6)	56.4 (9.6)<br>58.1 (9.4)	T2DM	Tablet	16,490	5
Yang 2010	America	46.6	46.2	56.8 (12.9)	63.1 (13.3)	Hypertension	Pill	579,851	6
Zeng 2010	America	52.7	39.4	60.1 (13.1)	67.4 (13.7)	Hypertension	Pill	4,525	9
Balu 2009	America	73.1	83.0<br>77.7	51.9 (10.5)	56.0 (9.4)<br>56.1 (10.6)	Cardiovascular disease	Tablet	8,988	7
Chapman 2009	America	53.9	51.5	53.9 (8.7)	53.9 (8.1)	Hypertension	Pill	5,456	7
Haupt 2009	Sweden	/	/	/	/	Asthma	Inhalant	48	4
Shaya 2009	America	36.3		Only numbers per age range are given		Hypertension	Pill	568	8
Brixner 2008	America	47.7	41.5	Only numbers per age range are given		Hypertension	Tablet	8,711	6
Cheong 2008	America	28.7	28.9	62.9 (13.5)	62.0 (13.5)	T2DM	Tablet	22,332	7
Dickson 2008	America	25.5	24.6	61.7 (15.9)	63.9 (15.1)	Hypertension	/	4,076	6
Dickson 2008	America	17.5	17.3	75.6 (7.1)	76.2 (7.3)	Hypertension	/	5,704	6

Hess 2008	America	43.1	43.1	62.1 (12.7)	62.9 (13.1)	Hypertension	Tablet or capsule	14,449	7
Jackson 2008	Switzerland	41.5	44.6	Only numbers per age range are given		Hypertension	Tablet	684	7
Pan 2008	America	55.5	56.1	52.4 (0.13)	54.4 (0.07)	Diabetes	Tablet	9,170	5
Patel 2008	America	57.5	44.4<br>52.2<br>42.5<br>44.7	61.4 (12.0)	62.8 (12.6)<br>63.1 (12.1)<br>62.1 (11.4)<br>64.4 (12.3)	Hypertension	Tablet	4,703	7
Gerbino 2007	America	/	/	/	/	Hypertension	Tablet	6,206	5
LaFleur 2006	America	80.7	75.6	55.9 (10.4)	57.8 (10.3)	Hyperlipidemia	/	825	7
Vanderpoel 2004	America	45.3<br>50.4	48.3<br>40.7	54.9 (11.4)<br>53.7 (10.6)	56.9 (11.3)<br>56.0 (9.7)	T2DM	Pill	2,637	5
Taylor 2003	America	50.0	50.0	52.0	54.0	Hypertension	Pill	5,732	7
Melikian 2002	America	49.5<br>50.1		62.5 (14.8)<br>67.0 (12.5)		Diabetes	Tablet	1,870<br>1,920	5

Abbreviations: FDC: fixed-dose combination; FEC: free-equivalent components; COPD: chronic obstructive pulmonary disease; T2DM: type 2 diabetes; LUTS: lower urinary tract symptoms; BPH: benign prostatic hyperplasia; AIDS: acquired immune deficiency syndrome

Table S3 Studies of Medication Adherence Measurement Using MPR or/and PDC

Study	Period of evaluation	Measurement method	Study group (FDC)	Control group (FEC)	Outcome	
					FDC	FEC
Mannino 2022	1 year	Mean PDC	FF/UMEC/VI	ICS + LAMA + LABA	Mean (median): 0.60 (0.74)<br>0.8 or above: 43.2%	Mean (median): 0.40 (0.32)<br>0.8 or above: 17.4%
	6 months				Mean (median): 0.66 (0.74)<br>0.8 or above: 46.5%	Mean (median): 0.48 (0.44)<br>0.8 or above: 22.3%
Bohm 2021	1 year	PDC: recipients were adherent with drug therapy if the PDC was 0.8 or above.	Sitagliptin/metformin	Sitagliptin + metformin	ATT (SE): 0.22 (0.02)	
	2 years				ATT (SE): 0.25 (0.02)	
	3 years				ATT (SE): 0.29 (0.03)	
Choi 2021	180 days<br>360 days<br>540 days	PDC: recipients were adherent with drug therapy if the PDC was 0.8 or above.	Rosuvastatin/OL, rosuvastatin/valsartan, rosuvastatin/telmisartan, atorvastatin/irbesartan	Rosuvastatin + OL, rosuvastatin + valsartan, rosuvastatin + telmisartan, atorvastatin + irbesartan	Mean: 0.902<br>0.8 or above: 85.3%<br>/<br>Mean (SD): 0.839 (0.233)<br>0.8 or above: 75.2%	Mean: 0.659<br>0.8 or above: 43.8%<br>/<br>Mean (SD): 0.578 (0.354)<br>0.8 or above: 41.1%
Rea 2021	1 year	PDC: recipients were adherent with drug therapy if the PDC was 0.75 or above.	Statin/Ezetimibe	Statin + Ezetimibe	0.75 above: 68.2%	0.75 above: 35.5%
Shirai 2021	1 year	PDC: recipients were adherent with drug therapy if the PDC was 0.8 or above.	PG/BB	PG + BB	Mean (SD): 0.791 (0.321)<br>0.8 or above: 69.60%	Mean (SD): 0.622 (0.380)<br>0.8 or above: 48.60%
Eisen 2020	2 years	MPR	Dutasteride/tamsulosin	AB + 5-α reductase inhibitor	0.8 or above: 63.1%	0.8 or above: 57.8%

Landeira 2020	1 year	MPR: recipients were adherent with drug therapy if the MPR was 0.8 or above.	AB/AM	AB + AM	Mean (SD): 0.488 (0.372)<br>0.8 or above: 34.2%	Mean (SD): 0.231 (0.284)<br>0.8 or above: 10%
Kim 2019	At least 1year, Maximum of 6 years	Mean MPR	ARB/CCB	ARB + CCB	mean (95% CI): 0.897 (0.893 - 0.900)	mean (95% CI): 0.872 (0.867 - 0.877)
Wang 2019	1 year	PDC: recipients were adherent with drug therapy if the PDC was 0.8 or above.	Single-pill triple combination	Dual-combination + third agent<br>Triple-pill free combination	Mean: 0.67	Mean: 0.37<br>Mean: 0.50
Ah 2019	/	MPR: recipients were adherent with drug therapy if the MPR was 0.8 or above.	ARB/CCB	ARB + CCB	Mean (SD): 0.8 (0.3)<br>0.8 or above: 68.5%	Mean (SD): 0.7 (0.3)<br>0.8 or above: 63.1%
Bramlage 2018	1 year	MPR: recipients were adherent with drug therapy if the MPR was 0.8 or above.	Ramipril/AML<br>Candesartan/AML	Ramipril + AML<br>Candesartan + AML	0.8 or above: 52.1%<br>0.8 or above: 84.6%	0.8 or above: 32.9%<br>0.8 or above: 58.2%
Ho 2018	Mean follow-up of 887.89 and 830.22 days	PDC: recipients were adherent with drug therapy if the PDC was 0.8 or above.	RAS inhibitor/ thiazide diuretic	RAS inhibitor + thiazide diuretic	Mean (SD): 0.5801 (0.3301)<br>0.8 or above: 35.4%	Mean (SD): 0.4696 (0.3652)<br>0.8 or above: 28.2%
Verma 2018	5 years	Median PDC	ACEI/thiazide diuretic, ARB/thiazide diuretic	ACEI + thiazide diuretic, ARB + thiazide diuretic	median (IQR): 0.70 (0.19–0.98)	median (IQR): 0.42 (0.11–0.91)
Bartlett 2017	6 months	MPR: recipients were adherent with drug therapy if the MPR was 0.8 or above.	Ezetimibe/statin	Ezetimibe + statin	Mean (95%CI): 0.97 (0.95, 0.99)<br>0.8 or above: 79.1%	Mean (95%CI): 0.99 (0.98, 1.01)<br>0.8 or above: 83.6%

Drake 2017	1 year	MPR: recipients were adherent with drug therapy if the MPR was 0.8 or above.	AB/AM	AB + AM	Mean (SD): 0.91 (0.52)<br>0.8 or above: 80.0%	AB: Mean (SD): 0.95 (0.37), 0.8 or above: 85.8%<br>OM: Mean (SD): 0.89 (0.31), 0.8 or above: 75.2%
Lauffenburger 2017	1 year	PDC: recipients were adherent with drug therapy if the PDC was 0.8 or above.	FDC	FEC	51.3%	42.1%
Schaffer 2017	2 years	Mean PDC	AML/atorvastatin	AML + atorvastatin	AML/atorvastatin FDC was associated with higher statin adherence among statin users and in individuals who started on lower AML doses.	
Yager 2017	/	MPR: recipients were adherent with drug therapy if the MPR was 0.9 or above.	EFV/FTC/TDF,<br>RPV/FTC/TDF,<br>EVG/COBI/FTC/TDF	FEC	Antiretroviral therapy: mean (SD): 0.815 (0.153)<br>0.9 or above: 34.6%<br>Non-antiretroviral therapy: mean (SD): 0.788 (0.156)	Antiretroviral therapy: mean (SD): 0.661 (0.211)<br>0.9 or above: 11.3%<br>Non-antiretroviral therapy: mean (SD): 0.808 (0.16)
Levi 2016	6 months	PDC: recipients were adherent with drug therapy if the PDC was 0.8 or above.	AML/OM	AML + OM	Mean (SD): 0.756 (0.320)<br>0.8 or above: 55.10%	Mean (SD): 0.414 (0.305)<br>0.8 or above: 15.90%
Saito 2016	1 year	PDC: recipients were adherent with drug therapy if the PDC was 0.8 or above.	Calblock/Olmetec	Calblock + Olmetec	Mean (SD): 0.710 (0.331)<br>0.8 or above: 58.1%	Mean (SD): 0.621 (0.362)<br>0.8 or above: 46.5%
	6 months				Mean (SD): 0.797 (0.280)<br>0.8 or above: 68.9%	Mean (SD): 0.720 (0.336)<br>0.8 or above: 59.5%
	3 months				Mean (SD): 0.870 (0.219)<br>0.8 or above: 77.6%	Mean (SD): 0.816 (0.279)<br>0.8 or above: 71.2%
Sutton 2016	Minimum follow-up of 60 days	PDC: recipients were adherent with drug therapy if the PDC was 0.8 or above.	FDC	FEC	Median (SD): 0.80 (0.25)<br>0.8 or above: 49.8%	Median (SD): 0.67 (0.28)<br>0.8 or above: 35.1%

Sutton 2016	Minimum follow-up of 60 days	MPR: recipients were adherent with drug therapy if the MPR was 0.8 or above.	FDC	FEC	0.8 or above: 90%	0.8 or above: 77.5%
Lokhandwala 2015	1 year	MPR: recipients were adherent with drug therapy if the MPR was 0.8 or above.	FDC	FEC	Mean (SD): 0.78 (0.2)<br>0.8 or above: 57.0%	Mean (SD): 0.76 (0.2)<br>0.8 or above: 50.7%
Machnicki 2015	1 year	PDC: recipients were adherent with drug therapy if the PDC was 0.8 or above.<br>MPR: recipients were adherent with drug therapy if the MPR was 0.8 or above.	AML/valsartan/HCTZ	AML + valsartan + HCTZ	Mean MPR: 0.8570<br>MPR≥80%: 72.9%<br>Mean PDC: 0.7380<br>PDC≥80%: 55.2%	Mean MPR: 0.7700 MPR≥80%: 57.5%<br>Mean PDC: 0.6660 PDC≥80%: 33.4%
Tennant 2015	Median follow-up of 22 and 14 months	MPR: recipients were adherent with drug therapy if the MPR was 0.9 or above. Determining patient compliance through computerized pharmacy refill records.	EFV/emtricitabine/TDF	ATV + ritonavir + emtricitabine/TDF	0.9 or above: 51.5%	0.9 or above: 61.6%
		MPR: recipients were adherent with drug therapy if the MPR was 0.9 or above. Determining patient compliance through self-reports.			0.9 or above: 85.4%	0.9 or above: 92.8%

Baggarly 2014	6 months	MPR: recipients were adherent with drug therapy if the MPR was 0.8 or above.	FDC	FEC	Mean (SD): 0.70 (0.26)<br>0.8 or above: 44%	Mean (SD): 0.68 (0.24)<br>0.8 or above: 40%
	1 year				Mean (SD): 0.37 (0.39)<br>0.8 or above: 23%	Mean (SD): 0.42 (0.37)<br>0.8 or above: 24%
Degli 2014	6 months	PDC: recipients were adherent with drug therapy if the PDC was 0.8 or above.	Switched from AML + OM to AML/OM	AML + OM	0.8 or above: 76%	0.8 or above: 61.5%
Hsu 2014	6 months	MPR: recipients were adherent with drug therapy if the MPR was 0.8 or above.	ARB/thiazide diuretic	ARB + thiazide diuretic	Mean (SD): 0.7020 (0.2932)<br>0.8 or above: 66.55%	Mean (SD): 0.7047 (0.2877)<br>0.8 or above: 63.86%
	1 year				Mean (SD): 0.5909 (0.3356)<br>0.8 or above: 52.58%	Mean (SD): 0.564 (0.3336)<br>0.8 or above: 46.73%
	1.5 year				Mean (SD): 0.5359 (0.3487)<br>0.8 or above: 46.3%	Mean (SD): 0.4846 (0.3385)<br>0.8 or above: 38.07%
	2 years				Mean (SD): 0.4988 (0.3525)<br>0.8 or above: 42.06%	Mean (SD): 0.4321 (0.3348)<br>0.8 or above: 32.45%
Tung 2014	Mean follow-up of 15.2 months	Mean PDC	AML/valsartan	ARB + CBB	Mean (SD): 0.8035 (0.2190)	Mean (SD): 0.7257 (0.2595)
Vittorino Gaddi 2014	1 year	MPR: recipients were adherent with drug therapy if the MPR was 0.8 or above.	FDC	FEC	Among prevalent patient cohorts: 0.8 or above: 68.54%	0.8 or above: 61.42%
					Among incident patient cohorts: 0.8 or above: 67.36%	0.8 or above: 30.87%

Wang 2014	1 year	MPR: recipients were adherent with drug therapy if the MPR was 0.8 or above.	Switched from ACEI + thiazide-type diuretic or ARB + thiazide-type diuretic to ACEI/thiazide-type diuretic or ARB/thiazide-type diuretic	ACEI + thiazide-type diuretic or ARB + thiazide-type diuretic	Mean: 0.688 (0.319)<br>0.8 or above: 18.64%	Mean (SD): 0.419 (0.327)
Xie 2014	1 year	Mean PDC	Single-pill triple combination	Dual-combination + third agent	0.8 or above: 55.31%	0.8 or above: 40.44%
				Triple-pill free combination		0.8 or above: 32.61%
Ferrario 2013	1 year	PDC: recipients were adherent with drug therapy if the PDC was 0.8 or above.	AML/OM	AML + ARB	Mean (SD): 0.63 (0.34)<br>0.8 or above: 44.12%	Mean (SD): 0.34 (0.34)<br>0.8 or above: 19.53%
			AML/BEN		Mean (SD): 0.55 (0.37)<br>0.8 or above: 36.46%	
Panjabi 2013	around 3 years	PDC: recipients were adherent with drug therapy if the PDC was 0.8 or above.	AML/ARB + HCTZ, ARB/HCTZ + AML	AML + HCTZ + ARB	0.8 or above: 25.8%	0.8 or above: 13.8%
			AML/ACEI + HCTZ, ACEI/HCTZ + AML	AML + HCTZ + ACEI	0.8 or above: 23.8%	0.8 or above: 16%
			BB/HCTZ + AML	AML + HCTZ + BB	0.8 or above: 28.4%	0.8 or above: 13.6%
Kauf 2012	Mean follow-up of 272 and 338 days	MPR: recipients were adherent with drug therapy if the MPR was 0.8 or above.	ABC/ lamivudine	ABC + lamivudine	Mean (SD): 0.88 (0.17)<br>0.8 or above: 77.2%	Mean (SD): 0.82 (0.20)<br>0.8 or above: 65.2%
Barner 2011	1 year	MPR: recipients were adherent with drug therapy if the MPR was 0.8 or above.	Switched from pioglitazone + metformin to pioglitazone/metformin	Pioglitazone + metformin	Mean (SD): 0.828 (0.182)<br>0.8 or above: 61.7%	Mean (SD): 0.76 (0.168)<br>0.8 or above: 48.3%

Baser 2011	1 year	PDC: recipients were adherent with drug therapy if the PDC was 0.8 or above.	Valsartan/AML	ARB + CCB	0.8 or above: 46.8%	0.8 or above: 40.9%
Breitscheidel 2011	1 year	MPR: recipients were adherent with drug therapy if the MPR was 0.8 or above.	ARB/HCTZ	ARB + HCTZ	0.8 or above: 70.1%	0.8 or above: 60.1%
			ARB/HCTZ + other anti-hypertensive	ARB + HCTZ + other anti-hypertensive	0.8 or above: 72.0%	0.8 or above: 58.6%
			ARB/AML	ARB + AML	0.8 or above: 60.8%	0.8 or above: 66.0%
			ARB/AML + other anti-hypertensive	ARB + AML + other anti-hypertensive	0.8 or above: 68.6%	0.8 or above: 66.4%
Kamat 2011	First 3 months of treatment	PDC: recipients were adherent with drug therapy if the PDC was 0.8 or above.	Simvastatin/ezetimibe, simvastatin/niacin, lovastatin/niacin	Simvastatin + ezetimibe, simvastatin + niacin, lovastatin + niacin	Mean (SD): 0.76 (0.26)	Mean (SD): 0.70 (0.27)
	Second 3 months of treatment				Mean (SD): 0.54 (0.40)	Mean (SD): 0.45 (0.40)
	1 year				Mean (SD): 0.50 (0.41)	Mean (SD): 0.41 (0.43)
Delea 2010	1 year	PDC: recipients were adherent with drug therapy if the PDC was 0.8 or above.	Levodopa/carbidopa/entacapone	Levodopa/carbidopa + entacapone	Levodopa therapy: Mean (SD): 0.93 (0.14), 0.8 or above: 87% Levodopa/carbidopa: Mean (SD): 0.94 (0.13), 0.8 or above: 88% Entacapone: Mean (SD): 0.93 (0.14), 0.8 or above: 87%	Levodopa therapy: Mean (SD): 0.71 (0.29), 0.8 or above: 51% Levodopa/carbidopa: Mean (SD): 0.94 (0.12), 0.8 or above: 87% Entacapone: Mean (SD): 0.90 (0.17), 0.8 or above: 81%

Hussein 2010	6 months	PDC: recipients were adherent with drug therapy if the PDC was 0.8 or above.	CCB/statin	CCB + statin	Naive (CCB)/ naive (statin): 0.8 or above: 41.6%	Naive (CCB)/ naive (statin): 0.8 or above: 39.5%
					Experienced (CCB)/ naive (statin) 0.8 or above: 52.2%	Experienced (CCB)/ naive (statin) 0.8 or above: 34.1%
					Naive (CCB)/ experienced (statin) 0.8 or above: 53.1%	Naive (CCB)/ experienced (statin) 0.8 or above: 34.8%
					Experienced (CCB)/ experienced (statin) 0.8 or above: 60.9%	Experienced (CCB)/ experienced (statin) 0.8 or above: 58.3%
	1 year				Naive (CCB)/ naive (statin): 0.8 or above: 32.2%	Naive (CCB)/ naive (statin): 0.8 or above: 28.1%
					Experienced (CCB)/ naive (statin) 0.8 or above: 46.5%	Experienced (CCB)/ naive (statin) 0.8 or above: 26.7%
					Naive (CCB)/ experienced (statin) 0.8 or above: 44.4%	Naive (CCB)/ experienced (statin) 0.8 or above: 27%
					Experienced (CCB)/ experienced (statin) 0.8 or above: 51%	Experienced (CCB)/ experienced (statin) 0.8 or above: 50.2%
Thayer 2010	Minimum follow-up of 182 days	Mean MPR	Switched from monotherapy to rosiglitazone/sulfonylurea	Switched from monotherapy to sulfonylurea + rosiglitazone	Mean: 0.87	Mean: 0.81
			Switched from dual therapy to rosiglitazone/sulfonylurea	Sulfonylurea + rosiglitazone	Mean: 0.87	Mean: 0.89
Yang 2010	6 months	Mean MPR	ARB/CCB, ARB/HCTZ, ACEI/HCTZ	ARB + CCB, ARB + HCTZ, ACEI + HCTZ	Mean: 0.728	Mean: 0.613

Zeng 2010	1 year	PDC: recipients were adherent with drug therapy if the PDC was 0.8 or above.	ARB/CCB	ARB + CCB	0.8 or above: 46%	0.8 or above: 35%
Balu 2009	1 year	MPR: recipients were adherent with drug therapy if the MPR was 0.8 or above.	Niacin extended-release/lovastatin	Niacin extended-release + lovastatin	Mean (SD): 0.54 (0.35)<br>0.8 or above: 34.2%	Mean (SD): 0.50 (0.35)<br>0.8 or above: 29.6%<br><br>Mean (SD): 0.47 (0.34)<br>0.8 or above: 25.9%
Chapman 2009	180 days	PDC: recipients were adherent with drug therapy if the PDC was 0.8 or above.	AML/atorvastatin	AML + statin	Mean (SD): 0.70 (0.28)<br>0.8 or above: 50.8%	Mean (SD): 0.65 (0.29)<br>0.8 or above: 44.3%
Haupt 2009	/	MPR: recipients were high adherence with drug therapy if the MPR was 0.8 or above.	Switched from LABA + ICS to FDC	LABA + ICS	0.8 or above: 62.5%	0.8 or above: 29.2%
Shaya 2009	At least 1 year	MPR: recipients were adherent with drug therapy if the MPR was 0.8 or above.	ACEI/HCTZ, ACEI/CCB	ACEI + HCTZ, ACEI + CCB	0.8 or above: 30.69%	0.8 or above: 21.58%
Brixner 2008	1 year	MPR: recipients were adherent with drug therapy if the MPR was 0.8 or above.	Valsartan/HCTZ	Valsartan + HCTZ	Persistent and MPR > 0.80: 36.27%	Persistent and MPR > 0.80: 12.66%

Cheong 2008	1 year	MPR: recipients were adherent with drug therapy if the MPR was 0.8 or above.	Glyburide/metformin, rosiglitazone/metformin, glipizide/metformin	Glyburide + metformin, rosiglitazone + metformin, glipizide + metformin	Mean (SD): 0.786 (0.196) switched from monotherapy: 0.8 or above: 78.5% switched from dual-therapy: 0.8 or above: 81.7%	Mean (SD): 0.772 (0.181) switched from monotherapy: 0.8 or above: 74.7% switched from dual-therapy: 0.8 or above: 80.9%
Dickson 2008	1 year	MPR	AML besylate/BEN HCl	DHP-CCB + ACEI	Compliance: 58.6%	Compliance: 48.1%
Dickson 2008	1 year	Mean MPR	AML besylate/BEN HCl	DHP calcium channel antagonist and ACEI	Mean (SD): 0.634 (0.294)	Mean (SD): 0.490 (0.234)
Hess 2008	1 year	MPR	ARB/HCTZ, ACEI /HCTZ, ACEI/ CCB	ARB + HCTZ, ACEI + HCTZ, ACEI + CCB	Compliance at month 12: 76.9%	Compliance at month 12: 54.4%
Jackson 2008	Until treatment discontinuati on	MPR: recipients were adherent with drug therapy if the MPR was 0.8 or above.	Valsartan/HCTZ + AML	Valsartan + HCTZ + AML	Mean: 0.731	Mean: 0.605
Pan 2008	180 days	MPR	Metformin/sulfonylurea	Metformin + sulfonylurea	People who took FDC had about 13% higher MPR than those who took the two pills alone.	
Patel 2008	180 days	PDC: recipients were adherent with drug therapy if the PDC was 0.8 or above. Mean PDC	AML/atorvastatin	AML + atorvastatin	0.8 or above: 67.7% Mean: 0.81	0.8 or above: 49.9% Mean: 0.72
				AML + other statin		0.8 or above: 40.4% Mean: 0.67
				other CCB + atorvastatin		0.8 or above: 46.9% Mean: 0.71
				other CCB + other statin		0.8 or above: 37.4% Mean: 0.64
Gerbino 2007	9-12 months	Mean MPR	AML/BEN	ACEI + DHP-CCB	Mean (SD): 0.88 (0.17)	Mean (SD): 0.69 (0.28)

LaFleur 2006	3 months	MPR: recipients were adherent with drug therapy if the MPR was 0.8 or above.<br>PDC: recipients were adherent with drug therapy if the PDC was 0.8 or above.	Extended-release niacin/lovastatin	Extended-release niacin + stain	Mean (SD): 0.88 (0.19)<br>0.8 or above: 72.5%<br>3rd Quarter: 0.8 or above: 16.1%<br>4rd Quarter: 0.8 or above: 12.2%	Mean (SD): 0.90 (0.22)<br>0.8 or above: 75.8%<br>3rd Quarter: 0.8 or above: 23.2%<br>4rd Quarter: 0.8 or above: 18.9%
Vanderpoel 2004	6 months	Mean MPR	Switched from monotherapy to rosiglitazone/metformin	Switched from monotherapy to metformin + rosiglitazone	Mean: 0.83	Mean: 0.67
			Switched from dual therapy to rosiglitazone/metformin	Switched from dual therapy to metformin + rosiglitazone	Mean: 0.82	Mean: 0.83
Taylor 2003	At least 1 year	Mean MPR	AML besylate/BEN HCl	ACEI + DHP-CCB	Mean: 0.808	Mean: 0.738
Melikian 2002	180 days	Mean MPR	Newly Treated Patients: glyburide/metformin	Metformin + glyburide	No significant differences in adherence	
			Previously Treated Patients: glyburide/metformin	Metformin + glyburide	Mean (95% CI): 0.77 (0.72-0.82)	Mean (95% CI): 0.54 (0.52-0.56)

Abbreviations: PDC: proportion of days covered; FF: fluticasone furoate; UMEC: umeclidinium; VI: vilanterol; ICS: inhaled corticosteroid; LAMA: long-acting muscarinic antagonist; LABA: long- acting β2 agonist; ATT: Average treatment effect on the treated subjects excess outcomes attributable to FDC with; SE: standard error; OL: olmesartan; SD: standard deviation; MPR: medication possession ratio; AB: α-blocker; AM: antimuscarinic; PG: Prostaglandin analog; BB: β-blocker; ARB: angiotensin receptor blocker; CCB: calcium channel blocker; RAS: renin-angiotensin system; ACEI: angiotensin converting enzyme inhibitors; FDC: fixed-dose combination; FEC: free-equivalent components; EFV: efavirenz; FTC: emtricitabine; TDF: tenofovir disoproxil fumarate; RPV: rilpivirine; EVG: elvitegravir; COBI: cobicistat; AML: amlodipine; ATV: atazanavir; DHP: dihydropyridine; BEN: benazepril; HCTZ: hydrochlorothiazide; ABC: abacavir sulfate

Table S4 the Newcastle-Ottawa Scale (Cohort Study)

Study	Item 1	Item 2	Item 3	Item 4	Item 5	Item 6	Item 7	Item 8	Total
Mannino 2022	1	1	1	1	2	1	1	1	9
Bohm 2021	1	1	1	1	1	1	1	0	7
Choi 2021	1	1	1	0	2	1	1	1	6
Rea 2021	1	1	1	1	2	1	1	1	5
Shirai 2021	0	1	1	0	1	1	1	1	9
Eisen 2020	0	1	1	1	0	1	1	1	6
Landeira 2020	1	1	1	0	0	1	1	0	8
Kim 2019	1	1	1	0	1	1	1	1	7
Wang 2019	0	1	1	0	0	1	1	0	4
Ah 2019	1	1	1	1	1	1	1	1	8
Bramlage 2018	1	1	1	1	0	1	1	0	6
Ho 2018	1	1	1	1	2	1	1	1	9
Verma 2018	1	1	1	1	2	1	1	1	9
Bartlett 2017	1	1	1	1	1	1	0	1	7
Drake 2017	1	1	1	1	0	1	1	1	7
Lauffenburger 2017	1	1	1	1	1	1	1	0	7
Schaffer 2017	1	1	1	1	1	1	1	0	7
Yager 2017	1	1	1	0	1	1	0	0	5
Levi 2016	1	1	1	0	0	1	0	0	4
Saito 2016	1	1	1	0	0	1	1	0	5
Sutton 2016	1	1	1	0	0	1	0	1	5
Sutton 2016	1	1	1	0	0	1	0	1	5
Lokhandwala 2015	1	1	1	1	1	1	1	1	8
Machnicki 2015	1	1	1	0	1	1	1	1	7
Tennant 2015	1	1	1	0	0	1	1	1	6
Baggarly 2014	1	1	1	1	1	1	1	1	8
Degli 2014	1	1	1	0	0	1	1	1	6
Hsu 2014	1	1	1	1	1	1	1	0	7
Tung 2014	1	1	1	0	1	1	1	1	7
Vittorino Gaddi 2014	1	1	1	0	0	1	1	1	6
Wang 2014	1	1	1	0	1	1	1	1	7
Xie 2014	1	1	1	0	1	1	1	1	7
Ferrario 2013	1	1	1	0	2	1	1	1	8
Panjabi 2013	1	1	1	0	1	1	1	1	7
Kauf 2012	1	1	1	1	1	1	1	0	7
Barner 2011	1	1	1	0	1	1	1	1	7
Baser 2011	1	1	1	1	2	1	1	1	9
Breitscheidel 2011	1	1	1	1	0	1	1	1	7
Kamat 2011	1	1	1	1	1	1	1	0	7
Delea 2010	1	1	1	1	2	1	1	1	9
Hussein 2010	1	1	1	1	2	1	1	0	8
Thayer 2010	1	1	1	0	1	1	0	0	5
yang 2010	1	1	1	1	1	1	0	0	6
zeng2010	1	1	1	1	2	1	1	1	9

Balu 2009	1	1	1	1	1	1	1	0	7
Chapman 2009	1	1	1	1	2	1	0	0	7
Haupt 2009	1	1	1	0	0	1	0	0	4
Shaya 2009	1	1	1	1	1	1	1	1	8
Brixner 2008	1	1	1	1	0	1	1	0	6
Cheong 2008	1	1	1	1	1	1	1	0	7
Dickson 2008	1	1	1	0	1	1	1	0	6
Dickson 2008	1	1	1	0	1	1	1	0	6
Hess 2008	1	1	1	0	2	1	1	0	7
Jackson 2008	1	1	1	1	1	1	1	0	7
Pan 2008	1	1	1	0	1	1	0	0	5
Patel 2008	1	1	1	1	2	1	0	0	7
Gerbino 2007	1	1	1	0	0	1	1	0	5
LaFleur 2006	1	1	1	1	1	1	1	0	7
Vanderpoel 2004	1	1	1	0	0	1	0	1	5
Taylor 2003	1	1	1	1	0	1	1	1	7
Melikian 2002	1	1	1	0	1	1	0	0	5

2 Supplementary Figures

Study or Subgroup	FDC Events	FDC Total	FEC Events	FEC Total	Weight	Risk Ratio M-H, Random, 95% CI	Year
**(a) T2DM patients**							
Lokhandwala 2015	7176	12590	5461	10771	2.8%	1.12 [1.10, 1.15]	2015
Gaddi 2014 (1)	20398	29759	11598	18883	2.8%	1.12 [1.10, 1.13]	2014
Gaddi 2014 (2)	1290	1915	213	690	2.5%	2.18 [1.94, 2.45]	2014
Cheong 2008 (1)	5639	7183	6630	8876	2.8%	1.05 [1.03, 1.07]	2008
Cheong 2008 (2)	316	387	4762	5886	2.7%	1.01 [0.96, 1.06]	2008
**Subtotal (95% CI)**		**51834**		**45106**	**13.5%**	**1.19 [1.10, 1.29]**	
Total events	34819		28664				
Heterogeneity: Tau² = 0.01; Chi² = 189.45, df = 4 (P < 0.00001); I² = 98%							
Test for overall effect: Z = 4.52 (P < 0.00001)							
**(b) CVD patients**							
Choi 2021 (1)	13255	14695	29052	44085	2.8%	1.37 [1.36, 1.38]	2021
Choi 2021 (2)	12329	14695	25481	44085	2.8%	1.45 [1.44, 1.47]	2021
Young 2019	13822	20175	12724	20175	2.8%	1.09 [1.07, 1.10]	2019
Bramlage 2018 (2)	1195	1413	5286	9082	2.8%	1.45 [1.41, 1.49]	2018
Ho 2018	4670	13176	1240	4392	2.7%	1.26 [1.19, 1.32]	2018
Bramlage 2018 (1)	5699	10938	19913	60525	2.8%	1.58 [1.55, 1.62]	2018
Bartlett 2016	2585	3092	4128	5219	2.8%	1.06 [1.04, 1.08]	2016
Levi 2016	1684	3057	1025	1833	2.7%	0.99 [0.94, 1.04]	2016
Machnicki 2015 (1)	1373	1884	1083	1884	2.7%	1.27 [1.21, 1.33]	2015
Machnicki 2015 (2)	1040	1884	629	1884	2.6%	1.65 [1.53, 1.78]	2015
Saito 2015 (1)	183	315	309	665	2.4%	1.25 [1.10, 1.42]	2015
Saito 2015 (2)	396	575	684	1150	2.6%	1.16 [1.08, 1.25]	2015
Saito 2015 (3)	520	670	1034	1452	2.7%	1.09 [1.03, 1.15]	2015
Baggarly 2014 (1)	169	383	195	487	2.2%	1.10 [0.94, 1.29]	2014
Baggarly 2014 (2)	88	383	117	487	1.8%	0.96 [0.75, 1.22]	2014
Hsu 2014	2226	5725	568	1623	2.6%	1.11 [1.03, 1.20]	2014
Xie 2014	4710	8516	3171	7842	2.8%	1.37 [1.32, 1.41]	2014
Ferrario 2013 (1)	4394	12051	1513	7748	2.7%	1.87 [1.77, 1.96]	2013
Ferrario 2013 (2)	2146	4864	1513	7748	2.7%	2.26 [2.14, 2.39]	2013
Baser 2011	1526	3259	3831	9369	2.7%	1.15 [1.10, 1.20]	2011
Breitscheidel 2011 (1)	3034	4328	260	432	2.6%	1.16 [1.08, 1.26]	2011
Breitscheidel 2011 (2)	2628	3650	357	609	2.7%	1.23 [1.15, 1.32]	2011
Breitscheidel 2011 (3)	441	725	492	745	2.6%	0.92 [0.85, 1.00]	2011
Breitscheidel 2011 (4)	574	837	748	1126	2.7%	1.03 [0.97, 1.10]	2011
Zeng 2010	1018	2213	809	2312	2.6%	1.31 [1.22, 1.41]	2010
Balu 2009 (1)	2270	6638	499	1687	2.6%	1.16 [1.07, 1.25]	2009
Balu 2009 (2)	2270	6638	172	663	2.4%	1.32 [1.15, 1.51]	2009
Chapman 2009	579	1139	1514	3417	2.7%	1.15 [1.07, 1.23]	2009
Patel 2008 (1)	538	795	367	735	2.6%	1.36 [1.24, 1.48]	2008
Patel 2008 (2)	538	795	470	1163	2.6%	1.67 [1.54, 1.82]	2008
Patel 2008 (3)	538	795	306	652	2.6%	1.44 [1.31, 1.59]	2008
Patel 2008 (4)	538	795	508	1358	2.6%	1.81 [1.66, 1.97]	2008
LaFleur 2006	177	244	263	347	2.5%	0.96 [0.87, 1.06]	2006
**Subtotal (95% CI)**		**151342**		**246981**	**86.5%**	**1.28 [1.21, 1.35]**	
Total events	89153		120261				
Heterogeneity: Tau² = 0.02; Chi² = 3023.15, df = 32 (P < 0.00001); I² = 99%							
Test for overall effect: Z = 8.78 (P < 0.00001)							
**Total (95% CI)**		**203176**		**292087**	**100.0%**	**1.27 [1.21, 1.34]**	
Total events	123972		148925				
Heterogeneity: Tau² = 0.03; Chi² = 4400.82, df = 37 (P < 0.00001); I² = 99%							
Test for overall effect: Z = 8.92 (P < 0.00001)							
Test for subgroup differences: Chi² = 2.16, df = 1 (P = 0.14), I² = 53.7%							

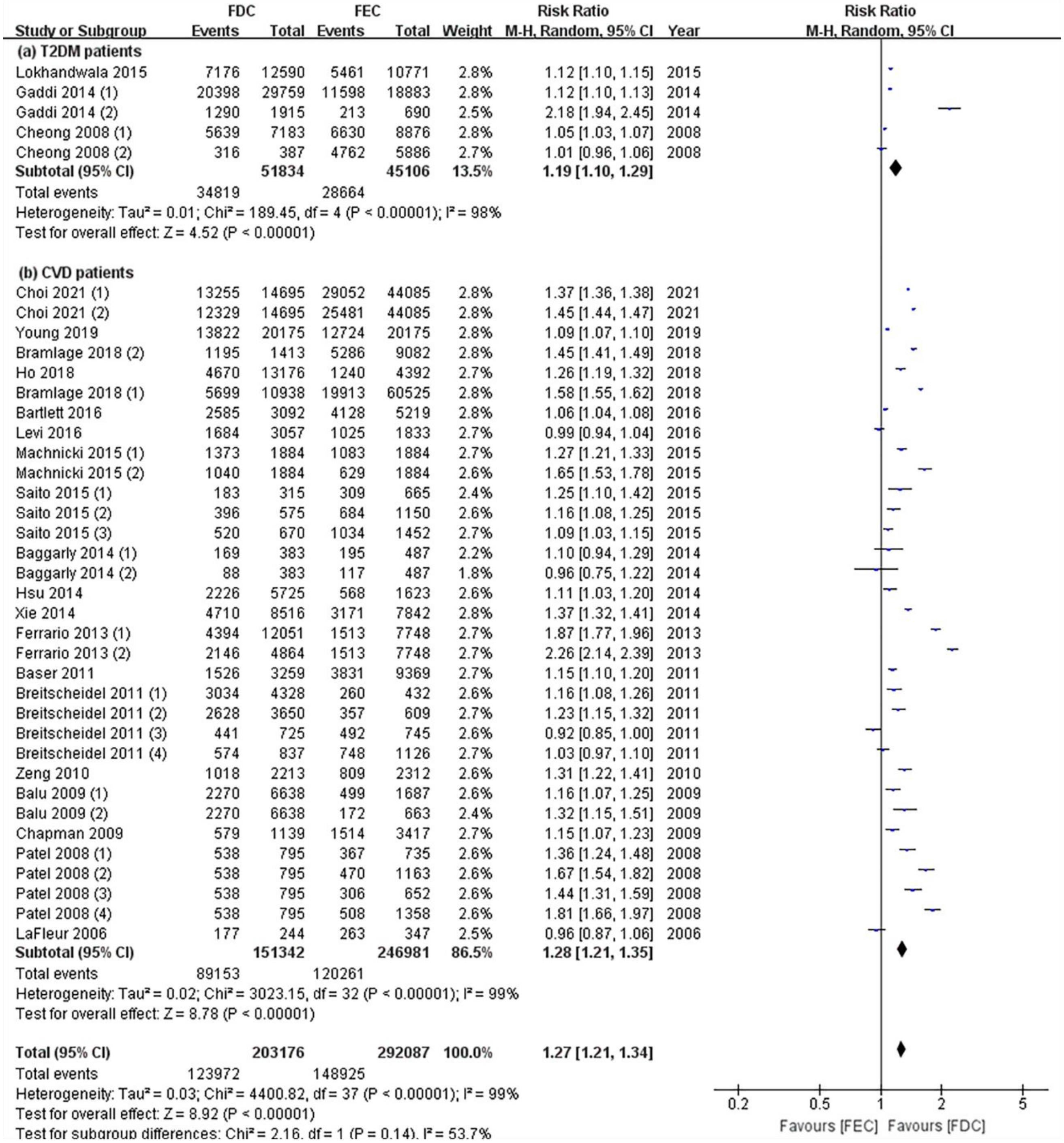

Figure S1 Meta-analysis of risk ratio for high medicine adherents (MPR or PDC ≥ 0.8) with T2DM and CVD. FDC: fixed-dose combination; FEC: free-equivalent components; MPR: medication possession ratio; PDC: proportion of days covered; T2DM: type 2 diabetes; CVD: cardiovascular diseases.

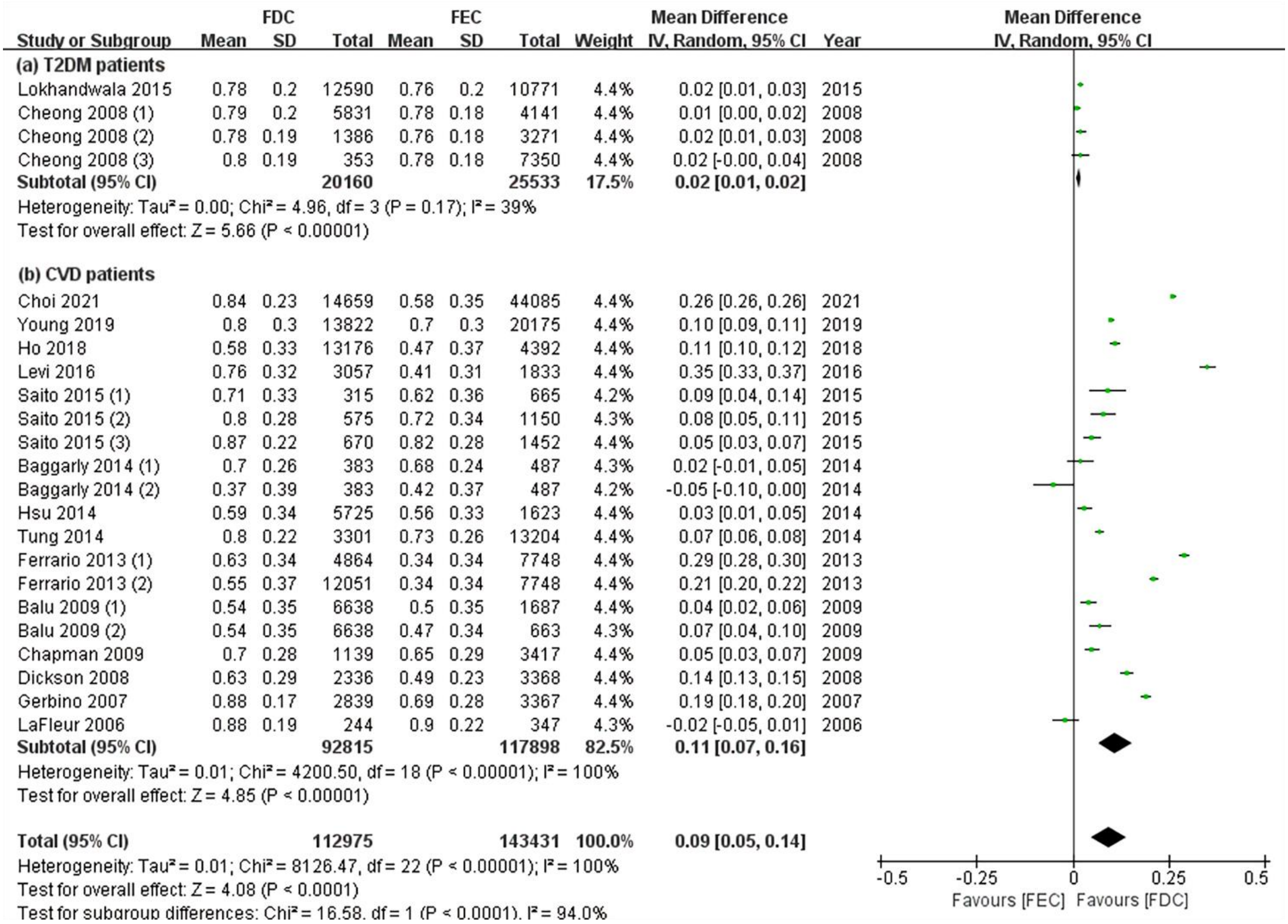

Study or Subgroup	FDC Mean	FDC SD	FDC Total	FEC Mean	FEC SD	FEC Total	Weight	Mean Difference IV, Random, 95% CI	Year
**(a) T2DM patients**									
Lokhandwala 2015	0.78	0.2	12590	0.76	0.2	10771	4.4%	0.02 [0.01, 0.03]	2015
Cheong 2008 (1)	0.79	0.2	5831	0.78	0.18	4141	4.4%	0.01 [0.00, 0.02]	2008
Cheong 2008 (2)	0.78	0.19	1386	0.76	0.18	3271	4.4%	0.02 [0.01, 0.03]	2008
Cheong 2008 (3)	0.8	0.19	353	0.78	0.18	7350	4.4%	0.02 [-0.00, 0.04]	2008
**Subtotal (95% CI)**			**20160**			**25533**	**17.5%**	**0.02 [0.01, 0.02]**	
Heterogeneity: Tau² = 0.00; Chi² = 4.96, df = 3 (P = 0.17); I² = 39%									
Test for overall effect: Z = 5.66 (P < 0.00001)									
**(b) CVD patients**									
Choi 2021	0.84	0.23	14659	0.58	0.35	44085	4.4%	0.26 [0.26, 0.26]	2021
Young 2019	0.8	0.3	13822	0.7	0.3	20175	4.4%	0.10 [0.09, 0.11]	2019
Ho 2018	0.58	0.33	13176	0.47	0.37	4392	4.4%	0.11 [0.10, 0.12]	2018
Levi 2016	0.76	0.32	3057	0.41	0.31	1833	4.4%	0.35 [0.33, 0.37]	2016
Saito 2015 (1)	0.71	0.33	315	0.62	0.36	665	4.2%	0.09 [0.04, 0.14]	2015
Saito 2015 (2)	0.8	0.28	575	0.72	0.34	1150	4.3%	0.08 [0.05, 0.11]	2015
Saito 2015 (3)	0.87	0.22	670	0.82	0.28	1452	4.4%	0.05 [0.03, 0.07]	2015
Baggarly 2014 (1)	0.7	0.26	383	0.68	0.24	487	4.3%	0.02 [-0.01, 0.05]	2014
Baggarly 2014 (2)	0.37	0.39	383	0.42	0.37	487	4.2%	-0.05 [-0.10, 0.00]	2014
Hsu 2014	0.59	0.34	5725	0.56	0.33	1623	4.4%	0.03 [0.01, 0.05]	2014
Tung 2014	0.8	0.22	3301	0.73	0.26	13204	4.4%	0.07 [0.06, 0.08]	2014
Ferrario 2013 (1)	0.63	0.34	4864	0.34	0.34	7748	4.4%	0.29 [0.28, 0.30]	2013
Ferrario 2013 (2)	0.55	0.37	12051	0.34	0.34	7748	4.4%	0.21 [0.20, 0.22]	2013
Balu 2009 (1)	0.54	0.35	6638	0.5	0.35	1687	4.4%	0.04 [0.02, 0.06]	2009
Balu 2009 (2)	0.54	0.35	6638	0.47	0.34	663	4.3%	0.07 [0.04, 0.10]	2009
Chapman 2009	0.7	0.28	1139	0.65	0.29	3417	4.4%	0.05 [0.03, 0.07]	2009
Dickson 2008	0.63	0.29	2336	0.49	0.23	3368	4.4%	0.14 [0.13, 0.15]	2008
Gerbino 2007	0.88	0.17	2839	0.69	0.28	3367	4.4%	0.19 [0.18, 0.20]	2007
LaFleur 2006	0.88	0.19	244	0.9	0.22	347	4.3%	-0.02 [-0.05, 0.01]	2006
**Subtotal (95% CI)**			**92815**			**117898**	**82.5%**	**0.11 [0.07, 0.16]**	
Heterogeneity: Tau² = 0.01; Chi² = 4200.50, df = 18 (P < 0.00001); I² = 100%									
Test for overall effect: Z = 4.85 (P < 0.00001)									
**Total (95% CI)**			**112975**			**143431**	**100.0%**	**0.09 [0.05, 0.14]**	
Heterogeneity: Tau² = 0.01; Chi² = 8126.47, df = 22 (P < 0.00001); I² = 100%									
Test for overall effect: Z = 4.08 (P < 0.0001)									
Test for subgroup differences: Chi² = 16.58, df = 1 (P < 0.0001), I² = 94.0%									

Figure S2 Meta-analysis of weighted mean difference in MPR or PDC in T2DM and CVD patients. FDC: fixed-dose combination; FEC: free-equivalent components; MPR: medication possession ratio; PDC: proportion of days covered; T2DM: type 2 diabetes; CVD: cardiovascular diseases.

Study or Subgroup	FDC Events	FDC Total	FEC Events	FEC Total	Weight	Risk Ratio M-H, Random, 95% CI	Year
**(a) Assessment at 12 months**							
Landeira 2020	270	790	21	209	1.4%	3.40 [2.24, 5.16]	2020
Bramlage 2018 (1)	5699	10938	19913	60525	3.6%	1.58 [1.55, 1.62]	2018
Bramlage 2018 (2)	1195	1413	5286	9082	3.6%	1.45 [1.41, 1.49]	2018
Bartlett 2016	2585	3092	4128	5219	3.6%	1.06 [1.04, 1.08]	2016
Lokhandwala 2015	7176	12590	5461	10771	3.6%	1.12 [1.10, 1.15]	2015
Machnicki 2015 (1)	1373	1884	1083	1884	3.5%	1.27 [1.21, 1.33]	2015
Machnicki 2015 (2)	1040	1884	629	1884	3.4%	1.65 [1.53, 1.78]	2015
Saito 2015	183	315	309	665	3.1%	1.25 [1.10, 1.42]	2015
Baggarly 2014	88	383	117	487	2.3%	0.96 [0.75, 1.22]	2014
Gaddi 2014 (1)	20398	29759	11598	18883	3.6%	1.12 [1.10, 1.13]	2014
Gaddi 2014 (2)	1290	1915	213	690	3.2%	2.18 [1.94, 2.45]	2014
Hsu 2014	2226	5725	568	1623	3.4%	1.11 [1.03, 1.20]	2014
Xie 2014	4710	8516	3171	7842	3.6%	1.37 [1.32, 1.41]	2014
Ferrario 2013 (1)	4394	12051	1513	7748	3.5%	1.87 [1.77, 1.96]	2013
Ferrario 2013 (2)	2146	4864	1513	7748	3.5%	2.26 [2.14, 2.39]	2013
Baser 2011	1526	3259	3831	9369	3.5%	1.15 [1.10, 1.20]	2011
Breitscheidel 2011 (1)	3034	4328	260	432	3.4%	1.16 [1.08, 1.26]	2011
Breitscheidel 2011 (2)	2628	3650	357	609	3.4%	1.23 [1.15, 1.32]	2011
Breitscheidel 2011 (3)	441	725	492	745	3.4%	0.92 [0.85, 1.00]	2011
Breitscheidel 2011 (4)	574	837	748	1126	3.5%	1.03 [0.97, 1.10]	2011
Zeng 2010	1018	2213	809	2312	3.4%	1.31 [1.22, 1.41]	2010
**Subtotal (95% CI)**		**111131**		**149853**	**69.6%**	**1.34 [1.23, 1.47]**	
Total events	63994		62020				
Heterogeneity: Tau² = 0.04; Chi² = 2259.74, df = 20 (P < 0.00001); I² = 99%							
Test for overall effect: Z = 6.49 (P < 0.00001)							
**(b) Assessment at 6 months**							
Choi 2021	13255	14695	29052	44085	3.6%	1.37 [1.36, 1.38]	2021
Levi 2016	1684	3057	1025	1833	3.5%	0.99 [0.94, 1.04]	2016
Saito 2015	396	575	684	1150	3.4%	1.16 [1.08, 1.25]	2015
Baggarly 2014	169	383	195	487	2.9%	1.10 [0.94, 1.29]	2014
Chapman 2009	579	1139	1514	3417	3.5%	1.15 [1.07, 1.23]	2009
Patel 2008 (3)	538	795	367	735	3.4%	1.36 [1.24, 1.48]	2008
Patel 2008 (4)	538	795	470	1163	3.4%	1.67 [1.54, 1.82]	2008
Patel 2008 (1)	538	795	306	652	3.3%	1.44 [1.31, 1.59]	2008
Patel 2008 (2)	538	795	508	1358	3.4%	1.81 [1.66, 1.97]	2008
**Subtotal (95% CI)**		**23029**		**54880**	**30.4%**	**1.32 [1.18, 1.47]**	
Total events	18235		34121				
Heterogeneity: Tau² = 0.03; Chi² = 276.78, df = 8 (P < 0.00001); I² = 97%							
Test for overall effect: Z = 4.80 (P < 0.00001)							
**Total (95% CI)**		**134160**		**204733**	**100.0%**	**1.33 [1.25, 1.42]**	
Total events	82229		96141				
Heterogeneity: Tau² = 0.03; Chi² = 2685.23, df = 29 (P < 0.00001); I² = 99%							
Test for overall effect: Z = 9.02 (P < 0.00001)							
Test for subgroup differences: Chi² = 0.09, df = 1 (P = 0.77), I² = 0%							

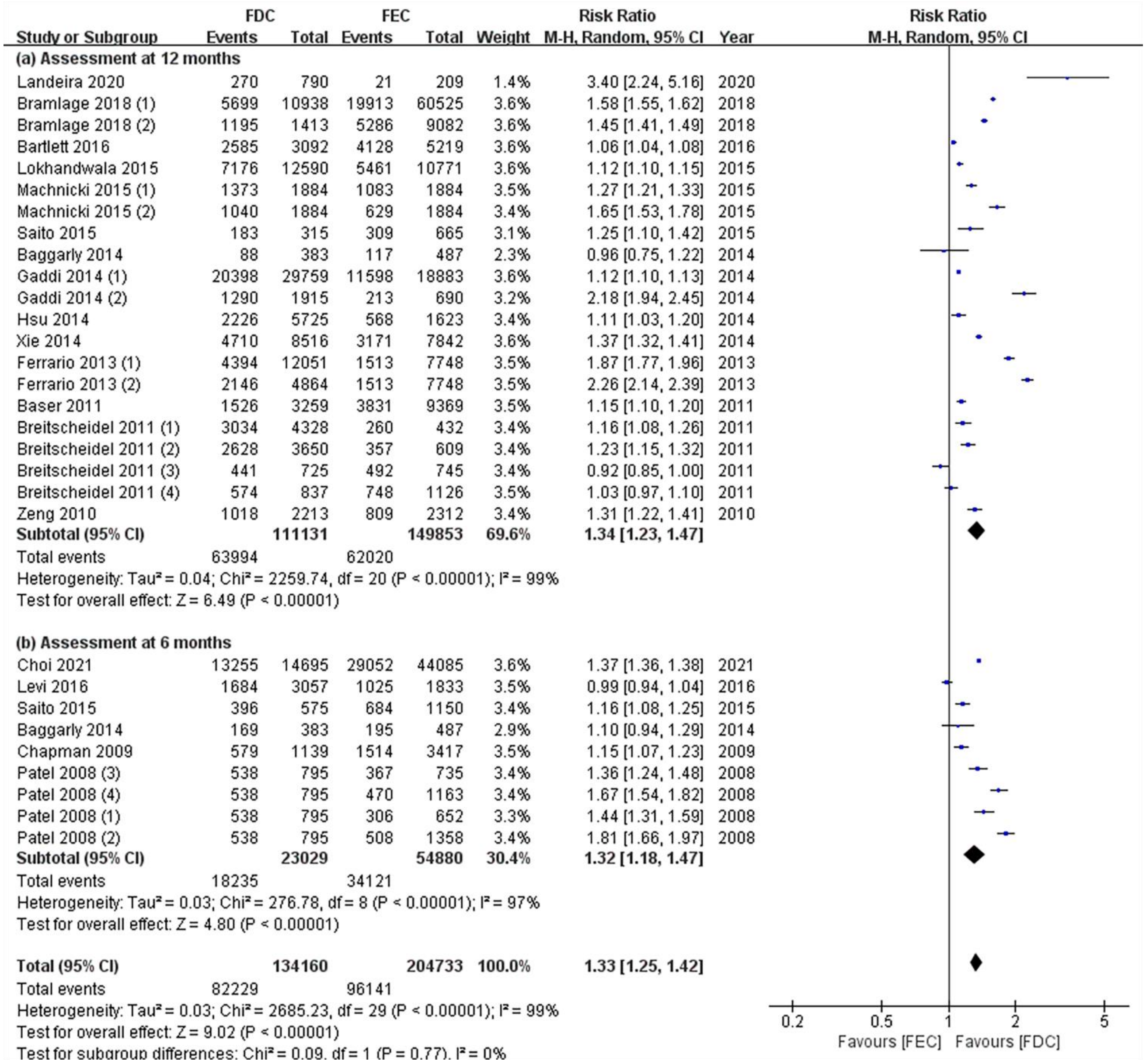

Figure S3 Meta-analysis of risk ratio for medicine high adherents (MPR or PDC ≥ 0.8) in 12 and 6 months. FDC: fixed-dose combination; FEC: free-equivalent components; MPR: medication possession ratio; PDC: proportion of days covered.

Study or Subgroup	FDC Mean	FDC SD	FDC Total	FEC Mean	FEC SD	FEC Total	Weight	Mean Difference IV, Random, 95% CI	Year
**(a) Assessment at 12 months**									
Landeira 2020	0.49	0.37	790	0.23	0.28	209	4.6%	0.26 [0.21, 0.31]	2020
Lokhandwala 2015	0.78	0.2	12590	0.76	0.2	10771	4.8%	0.02 [0.01, 0.03]	2015
Saito 2015	0.71	0.33	315	0.62	0.36	665	4.6%	0.09 [0.04, 0.14]	2015
Hsu 2014	0.59	0.34	5725	0.56	0.33	1623	4.8%	0.03 [0.01, 0.05]	2014
Baggarly 2014	0.37	0.39	383	0.42	0.37	487	4.5%	-0.05 [-0.10, 0.00]	2014
Ferrario 2013 (1)	0.63	0.34	4864	0.34	0.34	7748	4.8%	0.29 [0.28, 0.30]	2013
Ferrario 2013 (2)	0.55	0.37	12051	0.34	0.34	7748	4.8%	0.21 [0.20, 0.22]	2013
Balu 2009 (2)	0.54	0.35	6638	0.47	0.34	663	4.8%	0.07 [0.04, 0.10]	2009
Balu 2009 (1)	0.54	0.35	6638	0.5	0.35	1687	4.8%	0.04 [0.02, 0.06]	2009
Dickson 2008	0.63	0.29	2336	0.49	0.23	3368	4.8%	0.14 [0.13, 0.15]	2008
Cheong 2008 (1)	0.79	0.2	5831	0.78	0.18	4141	4.8%	0.01 [0.00, 0.02]	2008
Cheong 2008 (2)	0.78	0.19	1386	0.76	0.18	3271	4.8%	0.02 [0.01, 0.03]	2008
Cheong 2008 (3)	0.8	0.19	353	0.78	0.18	7350	4.8%	0.02 [-0.00, 0.04]	2008
Gerbino 2007	0.88	0.17	2839	0.69	0.28	3367	4.8%	0.19 [0.18, 0.20]	2007
**Subtotal (95% CI)**			**62739**			**53098**	**66.7%**	**0.10 [0.04, 0.15]**	
Heterogeneity: Tau² = 0.01; Chi² = 3327.03, df = 13 (P < 0.00001); I² = 100%									
Test for overall effect: Z = 3.50 (P = 0.0005)									
**(b) Assessment at 6 months**									
Levi 2016	0.76	0.32	3057	0.41	0.31	1833	4.8%	0.35 [0.33, 0.37]	2016
Saito 2015	0.8	0.28	575	0.72	0.34	1150	4.7%	0.08 [0.05, 0.11]	2015
Baggarly 2014	0.7	0.26	383	0.68	0.24	487	4.7%	0.02 [-0.01, 0.05]	2014
Chapman 2009	0.7	0.28	1139	0.65	0.29	3417	4.8%	0.05 [0.03, 0.07]	2009
**Subtotal (95% CI)**			**5154**			**6887**	**19.0%**	**0.13 [-0.05, 0.30]**	
Heterogeneity: Tau² = 0.03; Chi² = 641.50, df = 3 (P < 0.00001); I² = 100%									
Test for overall effect: Z = 1.42 (P = 0.16)									
**(c) Assessment at 3 months**									
Saito 2015	0.87	0.22	670	0.82	0.28	1452	4.8%	0.05 [0.03, 0.07]	2015
Kauf 2012	0.76	0.26	38847	0.7	0.27	3613	4.8%	0.06 [0.05, 0.07]	2012
LaFleur 2006	0.88	0.19	244	0.9	0.22	347	4.7%	-0.02 [-0.05, 0.01]	2006
**Subtotal (95% CI)**			**39761**			**5412**	**14.3%**	**0.03 [-0.00, 0.07]**	
Heterogeneity: Tau² = 0.00; Chi² = 20.82, df = 2 (P < 0.0001); I² = 90%									
Test for overall effect: Z = 1.72 (P = 0.09)									
**Total (95% CI)**			**107654**			**65397**	**100.0%**	**0.09 [0.05, 0.14]**	
Heterogeneity: Tau² = 0.01; Chi² = 4264.63, df = 20 (P < 0.00001); I² = 100%									
Test for overall effect: Z = 4.15 (P < 0.0001)									
Test for subgroup differences: Chi² = 4.13, df = 2 (P = 0.13), I² = 51.6%									

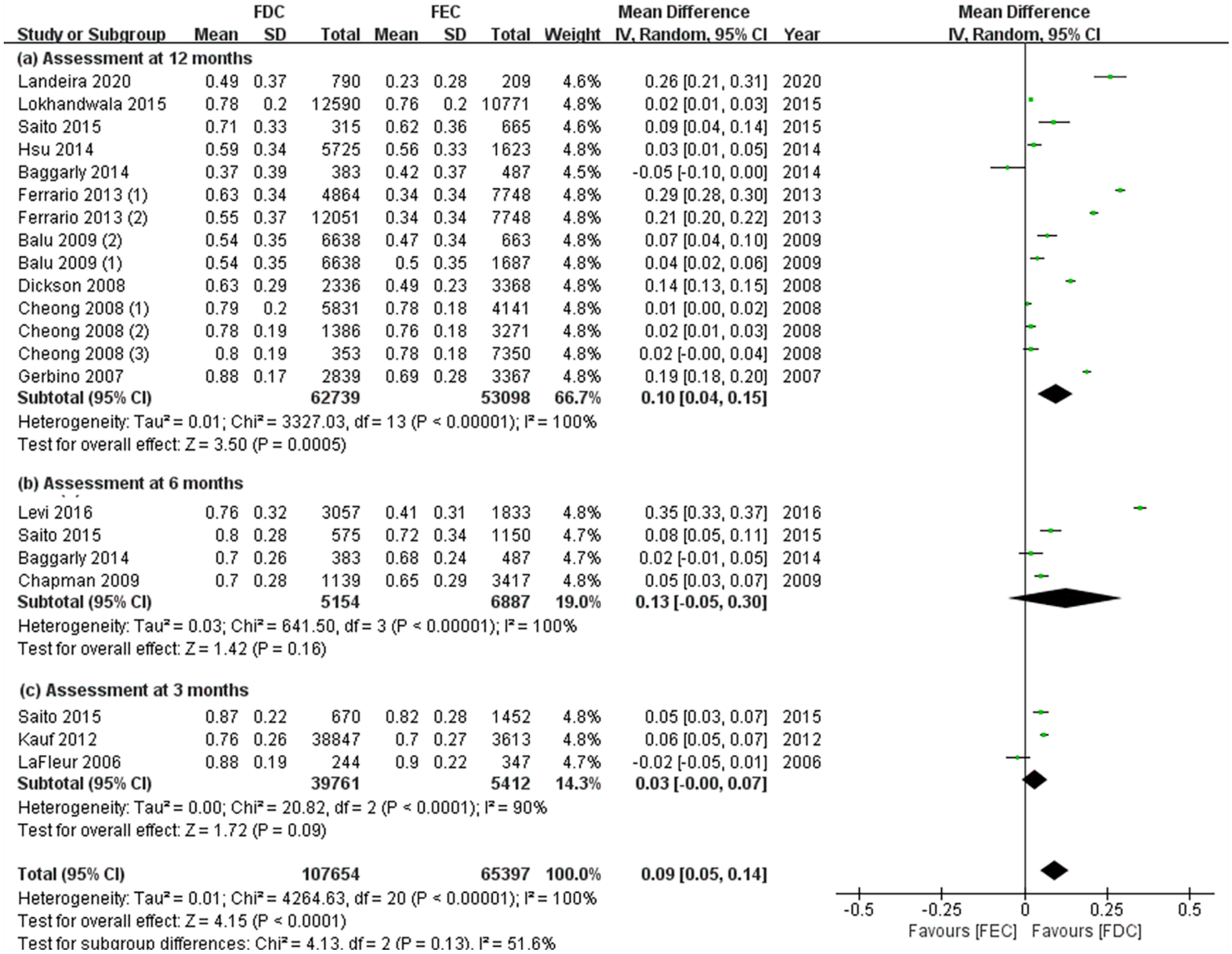

Figure S4 Meta-analysis of weighted mean difference in MPR or PDC in 12, 6 and 3months. FDC: fixed-dose combination; FEC: free-equivalent components; MPR: medication possession ratio; PDC: proportion of days covered.

Study or Subgroup	FDC Events	FDC Total	FEC Events	FEC Total	Weight	Risk Ratio M-H, Random, 95% CI	Year
**(a) MPR**							
Eisen 2020	54302	86057	32143	55610	2.5%	1.09 [1.08, 1.10]	2020
Landeira 2020	270	790	21	209	0.9%	3.40 [2.24, 5.16]	2020
Young 2019	13822	20175	12724	20175	2.5%	1.09 [1.07, 1.10]	2019
Bramlage 2018 (1)	5699	10938	19913	60525	2.5%	1.58 [1.55, 1.62]	2018
Bramlage 2018 (2)	1195	1413	5286	9082	2.5%	1.45 [1.41, 1.49]	2018
Yager 2017	377	1037	19	165	0.9%	3.16 [2.05, 4.86]	2017
Bartlett 2016	2585	3092	4128	5219	2.5%	1.06 [1.04, 1.08]	2016
Machnicki 2015	1373	1884	1083	1884	2.5%	1.27 [1.21, 1.33]	2015
Lokhandwala 2015	7176	12590	5461	10771	2.5%	1.12 [1.10, 1.15]	2015
Baggarly 2014 (1)	169	383	195	487	2.0%	1.10 [0.94, 1.29]	2014
Baggarly 2014 (2)	88	383	117	487	1.6%	0.96 [0.75, 1.22]	2014
Gaddi 2014 (1)	20398	29759	11598	18883	2.5%	1.12 [1.10, 1.13]	2014
Gaddi 2014 (2)	1290	1915	213	690	2.2%	2.18 [1.94, 2.45]	2014
Hsu 2014	2226	5725	568	1623	2.4%	1.11 [1.03, 1.20]	2014
Kauf 2012	502	650	1269	1947	2.5%	1.18 [1.12, 1.25]	2012
Breitscheidel 2011 (1)	3034	4328	260	432	2.4%	1.16 [1.08, 1.26]	2011
Breitscheidel 2011 (2)	2628	3650	357	609	2.4%	1.23 [1.15, 1.32]	2011
Breitscheidel 2011 (3)	441	725	492	745	2.4%	0.92 [0.85, 1.00]	2011
Breitscheidel 2011 (4)	574	837	748	1126	2.4%	1.03 [0.97, 1.10]	2011
Balu 2009 (1)	2270	6638	499	1687	2.4%	1.16 [1.07, 1.25]	2009
Balu 2009 (2)	2270	6638	172	663	2.1%	1.32 [1.15, 1.51]	2009
Cheong 2008 (1)	5639	7183	6630	8876	2.5%	1.05 [1.03, 1.07]	2008
Cheong 2008 (2)	316	387	4762	5886	2.5%	1.01 [0.96, 1.06]	2008
LaFleur 2006	177	244	263	347	2.3%	0.96 [0.87, 1.06]	2006
**Subtotal (95% CI)**		**207421**		**208128**	**54.1%**	**1.20 [1.13, 1.26]**	
Total events	128821		108921				
Heterogeneity: Tau² = 0.01; Chi² = 1758.73, df = 23 (P < 0.00001); I² = 99%							
Test for overall effect: Z = 6.62 (P < 0.00001)							
**(b) PDC**							
Choi 2021 (1)	13255	14695	29052	44085	2.5%	1.37 [1.36, 1.38]	2021
Choi 2021 (2)	12329	14695	25481	44085	2.5%	1.45 [1.44, 1.47]	2021
Ho 2018	4670	13176	1240	4392	2.5%	1.26 [1.19, 1.32]	2018
Sutton 2016	289	580	560	1594	2.3%	1.42 [1.28, 1.58]	2016
Levi 2016	1684	3057	1025	1833	2.5%	0.99 [0.94, 1.04]	2016
Saito 2015 (1)	183	315	309	665	2.2%	1.25 [1.10, 1.42]	2015
Saito 2015 (2)	396	575	684	1150	2.4%	1.16 [1.08, 1.25]	2015
Saito 2015 (3)	520	670	1034	1452	2.5%	1.09 [1.03, 1.15]	2015
Machnicki 2015	1040	1884	629	1884	2.4%	1.65 [1.53, 1.78]	2015
Xie 2014	4710	8516	3171	7842	2.5%	1.37 [1.32, 1.41]	2014
Ferrario 2013 (1)	4394	12051	1513	7748	2.5%	1.87 [1.77, 1.96]	2013
Ferrario 2013 (2)	2146	4864	1513	7748	2.5%	2.26 [2.14, 2.39]	2013
Baser 2011	1526	3259	3831	9369	2.5%	1.15 [1.10, 1.20]	2011
Zeng 2010	1018	2213	809	2312	2.4%	1.31 [1.22, 1.41]	2010
Chapman 2009	579	1139	1514	3417	2.4%	1.15 [1.07, 1.23]	2009
Patel 2008 (1)	538	795	367	735	2.4%	1.36 [1.24, 1.48]	2008
Patel 2008 (2)	538	795	470	1163	2.4%	1.67 [1.54, 1.82]	2008
Patel 2008 (3)	538	795	306	652	2.3%	1.44 [1.31, 1.59]	2008
Patel 2008 (4)	538	795	508	1358	2.4%	1.81 [1.66, 1.97]	2008
**Subtotal (95% CI)**		**84869**		**143484**	**45.9%**	**1.39 [1.31, 1.47]**	
Total events	50891		74016				
Heterogeneity: Tau² = 0.01; Chi² = 997.51, df = 18 (P < 0.00001); I² = 98%							
Test for overall effect: Z = 11.40 (P < 0.00001)							
**Total (95% CI)**		**292290**		**351612**	**100.0%**	**1.29 [1.23, 1.35]**	
Total events	179712		182937				
Heterogeneity: Tau² = 0.02; Chi² = 5419.94, df = 42 (P < 0.00001); I² = 99%							
Test for overall effect: Z = 10.07 (P < 0.00001)							
Test for subgroup differences: Chi² = 14.46, df = 1 (P = 0.0001), I² = 93.1%							

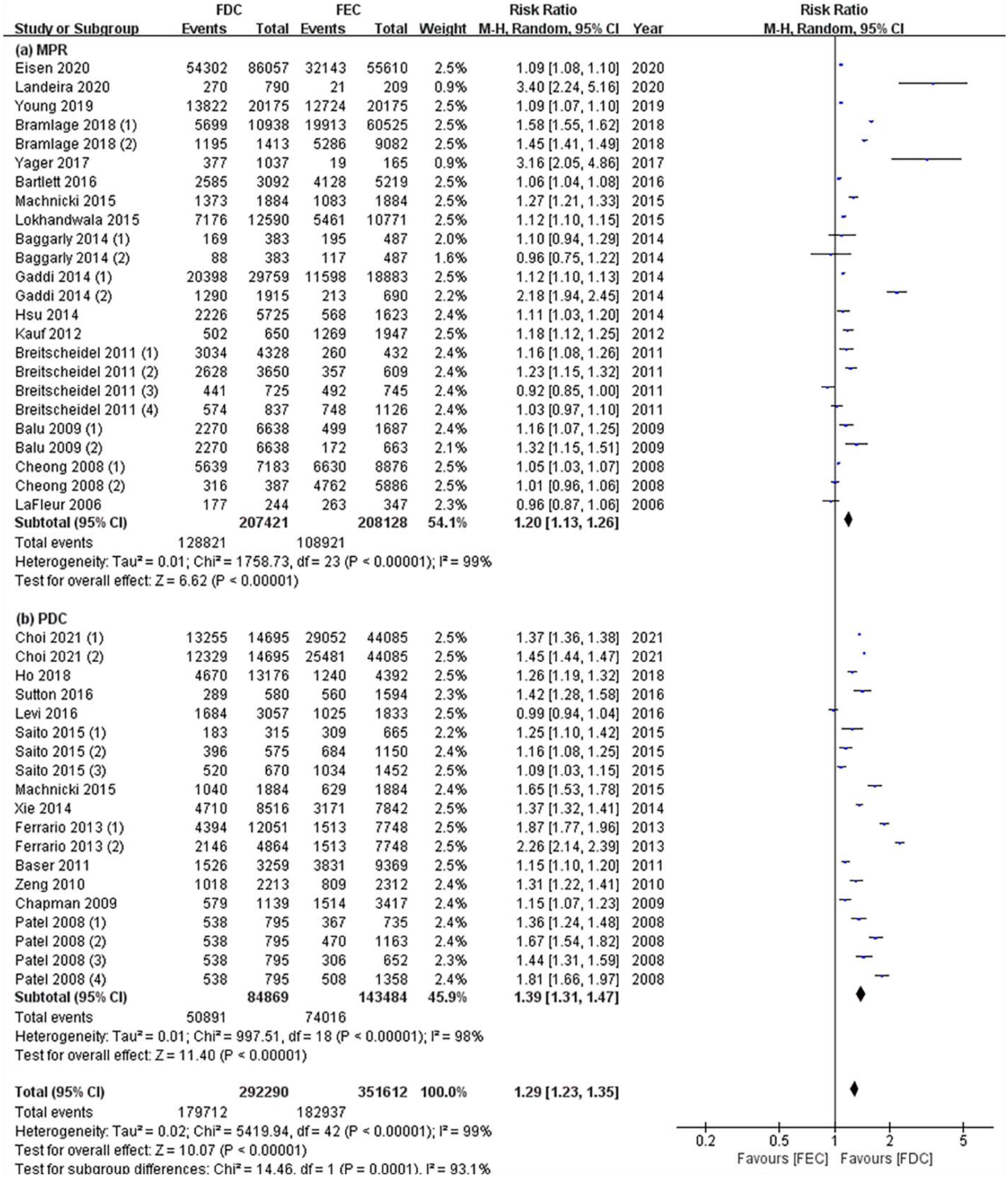

Figure S5 Meta-analysis of risk ratio for medicine high adherents in MPR and PDC subgroup. FDC: fixed-dose combination; FEC: free-equivalent components; MPR: medication possession ratio; PDC: proportion of days covered.

Study or Subgroup	FDC Mean	FDC SD	FDC Total	FEC Mean	FEC SD	FEC Total	Weight	Mean Difference IV, Random, 95% CI	Year
**(a) MPR**									
Landeira 2020	0.49	0.37	790	0.23	0.28	209	3.9%	0.26 [0.21, 0.31]	2020
Young 2019	0.8	0.3	13822	0.7	0.3	20175	4.0%	0.10 [0.09, 0.11]	2019
Yager 2017	0.82	0.15	1037	0.66	0.21	165	4.0%	0.16 [0.13, 0.19]	2017
Lokhandwala 2015	0.78	0.2	12590	0.76	0.2	10771	4.0%	0.02 [0.01, 0.03]	2015
Baggarly 2014 (1)	0.7	0.26	383	0.68	0.24	487	4.0%	0.02 [-0.01, 0.05]	2014
Baggarly 2014 (2)	0.37	0.39	383	0.42	0.37	487	3.8%	-0.05 [-0.10, 0.00]	2014
Hsu 2014	0.59	0.34	5725	0.56	0.33	1623	4.0%	0.03 [0.01, 0.05]	2014
Ferrario 2013 (1)	0.63	0.34	4864	0.34	0.34	7748	4.0%	0.29 [0.28, 0.30]	2013
Ferrario 2013 (2)	0.55	0.37	12051	0.34	0.34	7748	4.0%	0.21 [0.20, 0.22]	2013
Kauf 2012	0.88	0.17	650	0.82	0.2	1947	4.0%	0.06 [0.04, 0.08]	2012
Balu 2009 (1)	0.54	0.35	6638	0.5	0.35	1687	4.0%	0.04 [0.02, 0.06]	2009
Balu 2009 (2)	0.54	0.35	6638	0.47	0.34	663	4.0%	0.07 [0.04, 0.10]	2009
Cheong 2008 (1)	0.79	0.2	5831	0.78	0.18	4141	4.0%	0.01 [0.00, 0.02]	2008
Cheong 2008 (2)	0.78	0.19	1386	0.76	0.18	3271	4.0%	0.02 [0.01, 0.03]	2008
Cheong 2008 (3)	0.8	0.19	353	0.78	0.18	7350	4.0%	0.02 [-0.00, 0.04]	2008
Dickson 2008	0.63	0.29	2336	0.49	0.23	3368	4.0%	0.14 [0.13, 0.15]	2008
Gerbino 2007	0.88	0.17	2839	0.69	0.28	3367	4.0%	0.19 [0.18, 0.20]	2007
LaFleur 2006	0.88	0.19	244	0.9	0.22	347	4.0%	-0.02 [-0.05, 0.01]	2006
**Subtotal (95% CI)**			**78560**			**75554**	**72.0%**	**0.09 [0.05, 0.13]**	
Heterogeneity: Tau² = 0.01; Chi² = 3446.07, df = 17 (P < 0.00001); I² = 100%									
Test for overall effect: Z = 4.14 (P < 0.0001)									
**(b) PDC**									
Choi 2021	0.84	0.23	14659	0.58	0.35	44085	4.0%	0.26 [0.26, 0.26]	2021
Levi 2016	0.76	0.32	3057	0.41	0.31	1833	4.0%	0.35 [0.33, 0.37]	2016
Saito 2015 (1)	0.71	0.33	315	0.62	0.36	665	3.9%	0.09 [0.04, 0.14]	2015
Saito 2015 (2)	0.8	0.28	575	0.72	0.34	1150	4.0%	0.08 [0.05, 0.11]	2015
Saito 2015 (3)	0.87	0.22	670	0.82	0.28	1452	4.0%	0.05 [0.03, 0.07]	2015
Kamat 2011	0.76	0.26	38847	0.7	0.27	3613	4.0%	0.06 [0.05, 0.07]	2011
Chapman 2009	0.7	0.28	1139	0.65	0.29	3417	4.0%	0.05 [0.03, 0.07]	2009
**Subtotal (95% CI)**			**59262**			**56215**	**28.0%**	**0.13 [0.04, 0.23]**	
Heterogeneity: Tau² = 0.02; Chi² = 2215.41, df = 6 (P < 0.00001); I² = 100%									
Test for overall effect: Z = 2.67 (P = 0.008)									
**Total (95% CI)**			**137822**			**131769**	**100.0%**	**0.10 [0.06, 0.14]**	
Heterogeneity: Tau² = 0.01; Chi² = 8257.88, df = 24 (P < 0.00001); I² = 100%									
Test for overall effect: Z = 4.48 (P < 0.00001)									
Test for subgroup differences: Chi² = 0.74, df = 1 (P = 0.39), I² = 0%									

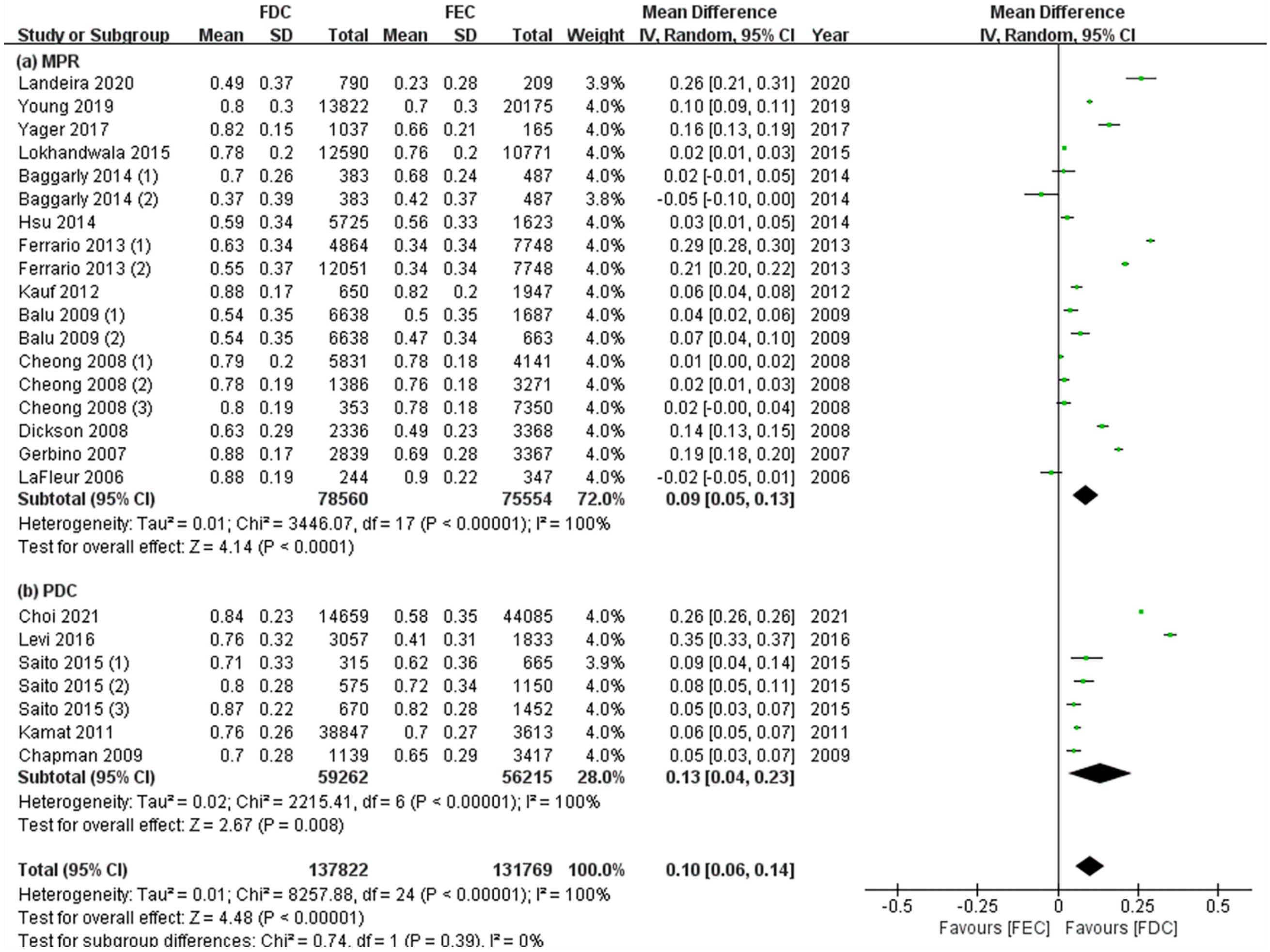

Figure S6 Meta-analysis of weighted mean difference in MPR and PDC subgroup. FDC: fixed-dose combination; FEC: free-equivalent components; MPR: medication possession ratio; PDC: proportion of days covered.

Study or Subgroup	FDC Events	FDC Total	FEC Events	FEC Total	Weight	Risk Ratio M-H, Random, 95% CI	Year
Choi 2021 (1)	13255	14695	29052	44085	3.0%	1.37 [1.36, 1.38]	2021
Choi 2021 (2)	12329	14695	25481	44085	3.0%	1.45 [1.44, 1.47]	2021
Eisen 2020	54302	86057	32143	55610	3.0%	1.09 [1.08, 1.10]	2020
Bramlage 2018 (1)	5699	10938	19913	60525	3.0%	1.58 [1.55, 1.62]	2018
Bramlage 2018 (2)	1195	1413	5286	9082	3.0%	1.45 [1.41, 1.49]	2018
Ho 2018	4670	13176	1240	4392	2.9%	1.26 [1.19, 1.32]	2018
Bartlett 2016	2585	3092	4128	5219	3.0%	1.06 [1.04, 1.08]	2016
Lokhandwala 2015	7176	12590	5461	10771	3.0%	1.12 [1.10, 1.15]	2015
Machnicki 2015 (1)	1373	1884	1083	1884	3.0%	1.27 [1.21, 1.33]	2015
Machnicki 2015 (2)	1040	1884	629	1884	2.9%	1.65 [1.53, 1.78]	2015
Xie 2014	4710	8516	3171	7842	3.0%	1.37 [1.32, 1.41]	2014
Baggarly 2014 (1)	169	383	195	487	2.4%	1.10 [0.94, 1.29]	2014
Baggarly 2014 (2)	88	383	117	487	1.9%	0.96 [0.75, 1.22]	2014
Gaddi 2014 (1)	20398	29759	11598	18883	3.0%	1.12 [1.10, 1.13]	2014
Gaddi 2014 (2)	1290	1915	213	690	2.7%	2.18 [1.94, 2.45]	2014
Hsu 2014	2226	5725	568	1623	2.9%	1.11 [1.03, 1.20]	2014
Ferrario 2013 (1)	4394	12051	1513	7748	2.9%	1.87 [1.77, 1.96]	2013
Ferrario 2013 (2)	2146	4864	1513	7748	2.9%	2.26 [2.14, 2.39]	2013
Kauf 2012	502	650	1269	1947	2.9%	1.18 [1.12, 1.25]	2012
Baser 2011	1526	3259	3831	9369	3.0%	1.15 [1.10, 1.20]	2011
Breitscheidel 2011 (1)	3034	4328	260	432	2.8%	1.16 [1.08, 1.26]	2011
Breitscheidel 2011 (2)	2628	3650	357	609	2.9%	1.23 [1.15, 1.32]	2011
Breitscheidel 2011 (3)	441	725	492	745	2.8%	0.92 [0.85, 1.00]	2011
Breitscheidel 2011 (4)	574	837	748	1126	2.9%	1.03 [0.97, 1.10]	2011
Zeng 2010	1018	2213	809	2312	2.9%	1.31 [1.22, 1.41]	2010
Balu 2009 (1)	2270	6638	499	1687	2.8%	1.16 [1.07, 1.25]	2009
Balu 2009 (2)	2270	6638	172	663	2.6%	1.32 [1.15, 1.51]	2009
Chapman 2009	579	1139	1514	3417	2.9%	1.15 [1.07, 1.23]	2009
Cheong 2008 (1)	5639	7183	6630	8876	3.0%	1.05 [1.03, 1.07]	2008
Cheong 2008 (2)	316	387	4762	5886	3.0%	1.01 [0.96, 1.06]	2008
Patel 2008 (1)	538	795	367	735	2.8%	1.36 [1.24, 1.48]	2008
Patel 2008 (2)	538	795	470	1163	2.8%	1.67 [1.54, 1.82]	2008
Patel 2008 (3)	538	795	306	652	2.8%	1.44 [1.31, 1.59]	2008
Patel 2008 (4)	538	795	508	1358	2.8%	1.81 [1.66, 1.97]	2008
LaFleur 2006	177	244	263	347	2.8%	0.96 [0.87, 1.06]	2006
**Total (95% CI)**		**265091**		**324369**	**100.0%**	**1.29 [1.22, 1.36]**	
Total events	162171		166561				

Heterogeneity: Tau² = 0.03; Chi² = 4920.06, df = 34 (P < 0.00001); I² = 99%

Test for overall effect: Z = 9.09 (P < 0.00001)

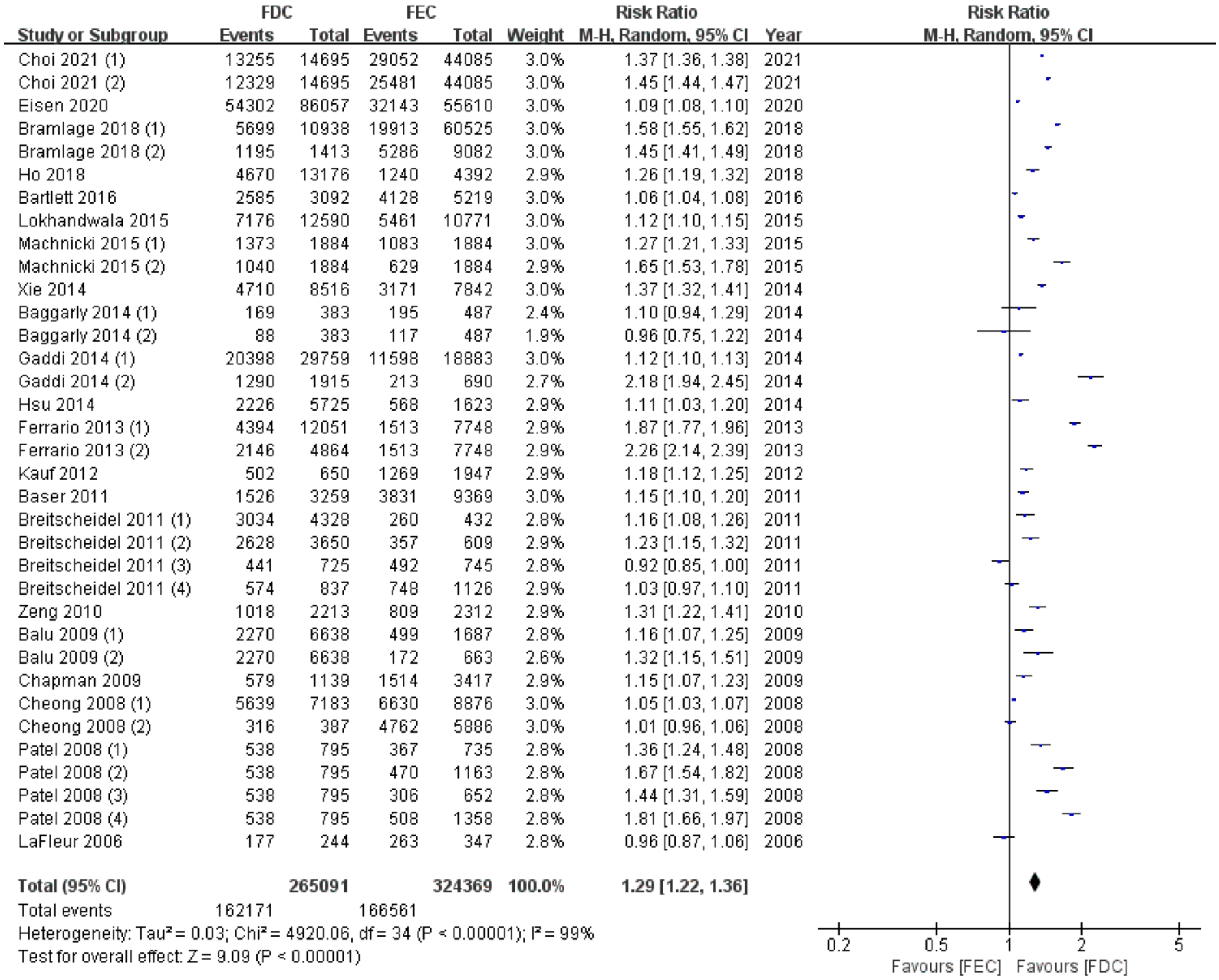

Figure S7 Meta-analysis of risk ratio for medicine high adherents (MPR or PDC ≥ 0.8) in sensitivity analysis. FDC: fixed-dose combination; FEC: free-equivalent components; MPR: medication possession ratio; PDC: proportion of days covered.

Study or Subgroup	FDC Mean	FDC SD	FDC Total	FEC Mean	FEC SD	FEC Total	Weight	Mean Difference IV, Random, 95% CI	Year
Choi 2021	0.84	0.23	14659	0.58	0.35	44085	6.7%	0.26 [0.26, 0.26]	2021
Lokhandwala 2015	0.78	0.2	12590	0.76	0.2	10771	6.7%	0.02 [0.01, 0.03]	2015
Hsu 2014	0.59	0.34	5725	0.56	0.33	1623	6.7%	0.03 [0.01, 0.05]	2014
Ferrario 2013 (1)	0.63	0.34	4864	0.34	0.34	7748	6.7%	0.29 [0.28, 0.30]	2013
Ferrario 2013 (2)	0.55	0.37	12051	0.34	0.34	7748	6.7%	0.21 [0.20, 0.22]	2013
Kauf 2012	0.88	0.17	650	0.82	0.2	1947	6.7%	0.06 [0.04, 0.08]	2012
Kamat 2011	0.76	0.26	38847	0.7	0.27	3613	6.7%	0.06 [0.05, 0.07]	2011
Balu 2009 (1)	0.54	0.35	6638	0.5	0.35	1687	6.7%	0.04 [0.02, 0.06]	2009
Balu 2009 (2)	0.54	0.35	6638	0.47	0.34	663	6.6%	0.07 [0.04, 0.10]	2009
Chapman 2009	0.7	0.28	1139	0.65	0.29	3417	6.7%	0.05 [0.03, 0.07]	2009
Cheong 2008 (1)	0.79	0.2	5831	0.78	0.18	4141	6.7%	0.01 [0.00, 0.02]	2008
Cheong 2008 (2)	0.78	0.19	1386	0.76	0.18	3271	6.7%	0.02 [0.01, 0.03]	2008
Cheong 2008 (3)	0.8	0.19	353	0.78	0.18	7350	6.7%	0.02 [-0.00, 0.04]	2008
Dickson 2008	0.63	0.29	2336	0.49	0.23	3368	6.7%	0.14 [0.13, 0.15]	2008
LaFleur 2006	0.88	0.19	244	0.9	0.22	347	6.6%	-0.02 [-0.05, 0.01]	2006
**Total (95% CI)**			**113951**			**101779**	**100.0%**	**0.08 [0.02, 0.15]**	

Heterogeneity: $Tau^2 = 0.01$; $Chi^2 = 7282.97$, df = 14 ($P < 0.00001$); $I^2 = 100\%$

Test for overall effect: Z = 2.69 (P = 0.007)

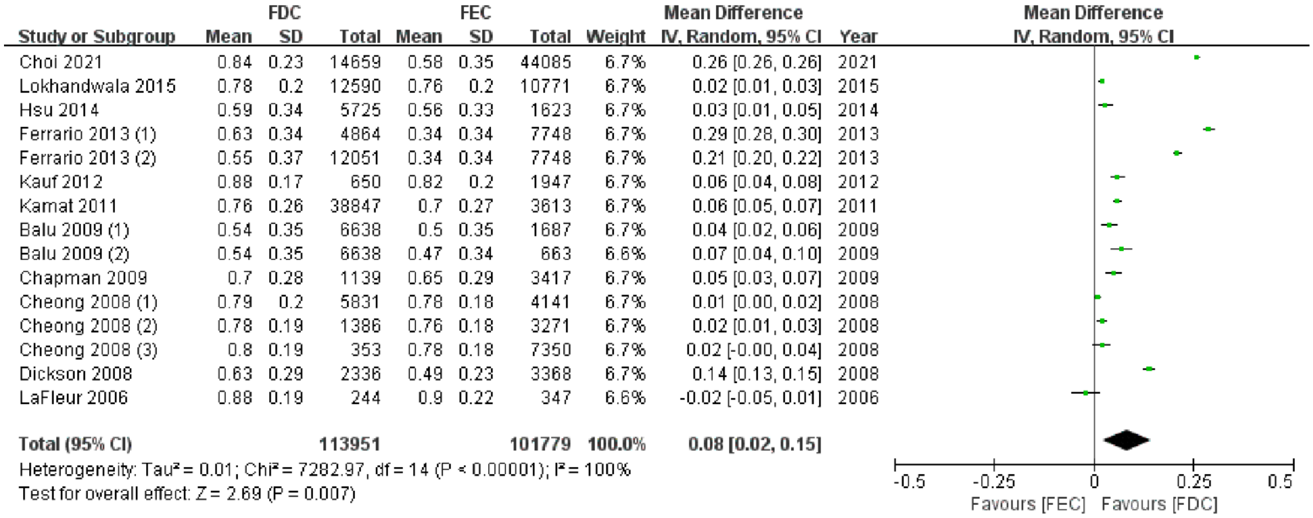

Figure S8 Meta-analysis of weighted mean difference in MPR or PDC in sensitivity analysis. FDC: fixed-dose combination; FEC: free-equivalent components; MPR: medication possession ratio; PDC: proportion of days covered; ITT: Intention-to-treat analysis.
